# Predicting Potential Range Shifts and Molecular Approaches in Four *Diolcogaster* Ashmead Species (Hymenoptera: Braconidae, Microgastrinae)

**DOI:** 10.1002/ece3.72547

**Published:** 2025-11-26

**Authors:** Asma Moeinadini, Mostafa Ghafouri Moghaddam, Diana Carolina Arias‐Penna, Seyed Massoud Madjdzadeh, Minoo Heidari Latibari, Buntika A. Butcher

**Affiliations:** ^1^ School of Biology, College of Science University of Tehran Tehran Iran; ^2^ Integrative Insect Ecology Research Unit, Department of Biology, Faculty of Science Chulalongkorn University Bangkok Thailand; ^3^ Independent researcher Bogotá Cundinamarca Colombia; ^4^ Department of Biology, Faculty of Sciences Shahid Bahonar University of Kerman Kerman Iran; ^5^ Center of Excellence in Entomology and Department of Biology, Faculty of Science Chulalongkorn University Bangkok Thailand; ^6^ Department of Plant Protection, Faculty of Agriculture Ferdowsi University of Mashhad Mashhad Iran

**Keywords:** bioclimatic variables, climate change, ecological differentiation, habitat suitability prediction, spatial distribution

## Abstract

*Diolcogaster* Ashmead is a genus of Microgastrinae (Hymenoptera: Braconidae) wasps with patchy distribution ranges. This study assessed and predicted the geographical distribution of four *Diolcogaster* species worldwide using field data and species distribution models (SDMs). The models projected the contemporary and future distributional ranges for the twenty‐first century. Nine informative environmental variables were employed to model the ecological niche with the MaxEnt model. Furthermore, a maximum likelihood phylogenetic estimation tree was reconstructed using IQ‐TREE, with the underlying data being *COI*‐based. Finally, PopART was used to perform a haplotype network analysis to assess the haplotype diversity and evolutionary distances of the four *Diolcogaster* species. The MaxEnt models showed excellent predictive performance (AUC > 0.9, TSS > 0.8) for all species. *Diolcogaster claritibia* occupied the widest and most suitable niches globally, followed by *D. mayae* , *D. alvearia* , and *D. spreta* . Molecular analyses supported the predicted models, indicating that *D. claritibia* 's adaptation to various habitats. Two environmental variables significantly influenced the distribution patterns of the four species. *Diolcogaster claritibia* and *D. mayae* are ecologically versatile, adapting to diverse habitats, elevations, and environmental conditions. Thus, their distribution ranges may extend beyond the previously documented limits. The four *Diolcogaster* species are currently predominantly found in temperate regions, preferring cooler climates. The model predicts that these species will expand into subtropical regions. This study offers a foundational theoretical framework for the practical rearing and strategic use of the wasps, as all Microgastrinae members are important biocontrol agents of caterpillar larvae.

## Graphical abstract

We modeled the global distributions of four *Diolcogaster* wasp species using MaxEnt and evaluated their ecological niches, phylogenetic relationships, and haplotype diversity. All species showed strong model performance, with *D. claritibia* exhibiting the broadest and most suitable habitats. Environmental variables strongly shaped species ranges, revealing that *D. claritibia* and *D. mayae* are highly adaptable across diverse climates and elevations. Although currently concentrated in temperate zones, all four species are predicted to expand into subtropical regions under future climate scenarios. These findings highlight the ecological flexibility and biocontrol potential of *Diolcogaster* wasps and provide an integrated ecological and evolutionary framework that supports their strategic rearing and targeted use in pest management.

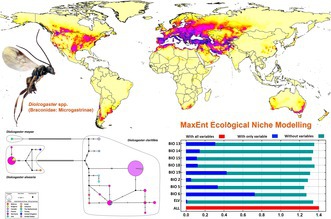

## 1 Introduction

The climate of Earth has undergone changes in the past and is expected to do so again in the future (Hayhoe et al. 2017; Rubenstein et al. 2023; Drake et al. 2025). The impact of climate change on insects is particularly pronounced. Projections indicate a significant decline in insect abundance, with a predicted decrease of nearly 50% over the next decade. Furthermore, a 27% decline in species richness within insect assemblages is projected (e.g., Dalton et al. 2023; Outhwaite et al. 2022; Harvey et al. 2023). These declines are expected to occur in environments that have experienced higher rates of historical climate warming. In contrast, less‐disturbed habitats have experienced more moderate climatic changes over the past decade (Trew and Maclean 2021; Outhwaite et al. 2022). These shifts are a direct consequence of changes in temperature, precipitation, and the frequency of extreme weather events. These factors have the capacity to modify the availability of resources, reproductive timing, and survival conditions for insects (Parmesan and Yohe 2003; Menéndez et al. 2007; Skendžić et al. 2021). These climatic alterations also profoundly affect the distribution patterns of various species of insects, including parasitoid wasps and their hosts (e.g., Li et al. 2022; Ghafouri Moghaddam and Butcher 2023; Ramos Aguila et al. 2023). As climate conditions shift, parasitoid wasps may experience positive or negative impacts. In certain instances, the proliferation of parasitoids is exacerbated by the augmented availability of their hosts, which frequently manifest as pest populations that thrive under warmer conditions (e.g., Hance et al. 2007; Ramos Aguila et al. 2023). However, in other cases, these wasps may experience difficulty in adapting to the changing environment, which could potentially result in a decline in their numbers and effectiveness in regulating pest populations (Furlong and Zalucki 2017).

It is imperative to acknowledge the pivotal role these dynamics play in the effective functioning of biological control programs and integrated pest management (Kogan 1998; Peshin and Dhawan 2009; Tokpah et al. 2016). In instances where climate change results in an increase in agricultural pest species, there is a corresponding rise in the potential for natural enemies, such as parasitoid wasps, to proliferate (e.g., Furlong and Zalucki 2017; Skendžić et al. 2021; Galli et al. 2024). A substantial body of research has demonstrated a positive correlation between the abundance of natural enemies and the rise in pest populations. The successful execution of parasitoid reproductive cycles is contingent upon the increased availability of host species (e.g., Spahn and Lill 2022). In turn, the efficiency of these natural enemies in regulating pest populations is influenced by their ability to cope with changing environmental conditions, synchronize with pest life cycles, and retain populations of endosymbionts essential to the parasitoids (e.g., Hance et al. 2007; Thomson et al. 2010; Tougeron et al. 2019; Spahn and Lill 2022; Heidari Latibari et al. 2020, 2022, 2023, 2025; Heidari Latibari, Moravvej, et al. 2022). Therefore, it is critical to comprehend the complex interplay between climate change, pest dynamics, and their natural enemies to develop sustainable pest management strategies in a warming world (Castex et al. 2018; Skendžić et al. 2021).

The braconid microgastrine parasitoid wasp (Hymenoptera: Ichneumonoidae) is one of the most critical parasitoid wasps in most terrestrial ecosystems worldwide (Whitfield et al. 2018; Ghafouri Moghaddam et al. 2019; Fernandez‐Triana et al. 2020; Ghafouri Moghaddam, Fernandez‐Triana, and Ward 2025; Ghafouri Moghaddam, Quicke, et al. 2025). However, extant data concerning the distribution, natural history, biology, and ecology of most species remain limited despite the implementation of extensive research initiatives (e.g., Shaw 2012; Valerio and Whitfield 2015; Arias‐Penna et al. 2013, 2019; Ghafouri Moghaddam et al. 2021; Žikić et al. 2024). In reality, there are only a few documented occurrences for the majority of species. While certain taxa exhibit extensive range sizes, substantial gaps in geographic localities often persist, encompassing diverse landscapes with different floras and ecologies (e.g., De la Pérez et al. 2020). Despite the prevalence of species revisions that address subtle morphological variations, there is an absence of substantiation for the ecological differentiation of species across wide geographic ranges (e.g., Fernández‐Triana et al. 2014; Arias‐Penna et al. 2019; Ghafouri Moghaddam et al. 2018, 2019). This is a salient consideration, as regional specialization may initially drive population differentiation and speciation, or may contribute to the development of broad floral preferences within a cryptic species. It has been proposed that populations at allopatric extremes can adapt to local environmental conditions, as opposed to speciation driven by host shifts, which appear to be nearly universal among microgastrines (e.g., Dupas et al. 2008; Gitau et al. 2007, 2010; Branca et al. 2011; Kaiser et al. 2015). The species of the wasp subfamily Microgastrinae are distinguished by their specific host preferences. Many species in this subfamily exhibit either sparse distributions or disjunct distributions, which can lead to population specialization through synchronization with dominant regional resources (e.g., Kaiser et al. 2015). For this reason, these wasps may serve as an ideal test case for understanding potential ecological differentiation across their distributions. This has the potential to stimulate additional research into the biology of specific localities at the extremes of their geographical distributions.

Species distribution models (SDMs), also referred to as ecological niche models (ENMs), have been identified as practical tools for predicting the impact of climate change on species (Guisan et al. 2017). SDMs use records of the occurrence of insects and plants, and environmental data to predict the habitat in which the target species is likely to be present (Sofaer et al. 2017; Norberg et al. 2019). A retrospective review identified over 35 methods for generating SDMs (Urbina‐Cardona et al. 2019). However, given the plethora of available models, it remains uncertain which possess the highest predictive accuracy, as each model has its own advantages and disadvantages (Guisan et al. 2017). Consequently, it is not possible to employ a single model to accurately predict the distribution of all species. Since 2006, researchers have underscored the importance of comparing SDMs that employ different modeling methods (Urbina‐Cardona et al. 2019). Utilizing multiple models simultaneously is recommended to facilitate a more comprehensive evaluation of which model most accurately reflects the species' distribution (Norberg et al. 2019). The most common combination on a global scale involves the maximum entropy (MaxEnt) model in conjunction with other SDMs, including the generalized linear model (GLM), random forest (RF), and generalized boosting model (GBM) (Norberg et al. 2019).

Previously studies have used ecological niche modeling to project the distributions of microgastrine species for which there is limited or no data for specific areas (De la Pérez et al. 2020; Ghafouri Moghaddam and Butcher 2023). However, an extensive survey of the bioecology of this group across varying climates, seasons, and temperatures remains to be conducted. In light of the pervasive climate change that is currently taking place on a global scale (Hayhoe et al. 2017; Rubenstein et al. 2023; Drake et al. 2025), meticulous studies on the ecology and bionomics of parasitoid wasps, particularly those belonging to the Microgastrinae, are indispensable. These wasps play a pivotal role in biological control and integrated pest management programs, underscoring their ecological significance (e.g., Whitfield et al. 2018; Ghafouri Moghaddam et al. 2019; Ghafouri Moghaddam, Tomlinson, et al. 2022). Compiling data on biodiversity, distribution, and ecology will serve to inform the development of guidelines for effectively controlling butterfly and moth pests (Macro‐ and Microlepidoptera). Furthermore, geographic information systems (GIS) represent a rapidly advancing technology that integrates graphical features with environmental data related to parasitoid wasps (De la Pérez et al. 2020; Ghafouri Moghaddam and Butcher 2023). This integration enables researchers to conduct more precise evaluations of species distribution and bioecology.

## 2 Material and Methods

### 2.1 Ecological Niche Modeling

The analysis incorporated four species of *Diolcogaster* Ashmead (Braconidae: Microgastrinae) that are distributed in the Western Palaearctic: *D. alvearia* (Fabricius), *D. claritibia* (Papp), *D. mayae* (Shestakov), and *D. spreta* (Marshall). All available occurrence points (locality and coordinates) for these species were used to ensure sufficient occurrence data for modeling. The information was derived from Ghafouri Moghaddam et al. (2019). Additionally, the checklist of world species of Microgastrinae parasitoid wasps (Fernandez‐Triana et al. 2020), the website Microgastrinae Wasps of the World (Ghafouri Moghaddam, Fernandez‐Triana, and Ward 2025; http://www.microgastrinae.myspecies.info/), the Global Biodiversity Information Facility (GBIF) (https://www.gbif.org/), and Taxapad (Yu et al. 2016) were consulted for global distribution information. To characterize environmental variation, climatic layers from the WorldClim data archive (Hijmans et al. 2005; https://www.worldclim.org/) with a spatial resolution of 10 arcminutes were utilized. The WorldClim database contains a total of 19 bioclimatic variables, and is a continuous dataset describing annual trends, seasonality, and some extreme conditions of temperature and precipitation conditions. To circumvent overfitting models to excessively high‐dimensional environmental spaces, dimensionality was reduced to nine principal components (Table 1). Consequently, these variables were selected based on their primary impact on the distribution of the four *Diolcogaster* species. The following bioclimatic (BIO) variables were incorporated into the analysis: mean diurnal range (mean of monthly temp [max temp‐min temp]) (BIO2), maximum temperature of the warmest month (BIO5), minimum temperature of the coldest month (BIO6), precipitation of the wettest month (BIO13), precipitation of the driest month (BIO14), precipitation seasonality (BIO15), precipitation of the warmest quarter (1/4 of the year or 3‐month period) (BIO18), and precipitation of the coldest quarter (BIO19). The environmental variable of elevation (ELV) is absent from the WorldClim database. Consequently, it was derived from the Digital Elevation Model (DEM) at an equivalent resolution (10 arcminutes) to ensure compatibility (Table 1). The nine principal components were generated within a geographic extent that included the occurrence points and an additional area presumed to be accessible to the species, based on the type of terrestrial ecoregion where the points were located (Olson et al. 2001).

**TABLE 1:** List of the bioclimatic variables used to predict the potential geographic distribution of four species of *Diolcogaster* Ashmead (Braconidae: Microgastrinae): *D. alvearia* (Fabricius), *D. claritibia* (Papp), *D. mayae* (Shestakov), and *D. spreta* (Marshall). The variables are derived from minimum, maximum, and mean temperature, as well as mean precipitation values.

Code	Bioclimatic variables	Units
BIO2	Mean diurnal range (mean of monthly temp (max temp–min temp))	°C
BIO5	Maximum temperature during the warmest month	°C
BIO6	Minimum temperature during the coldest month	°C
BIO13	Precipitation during the wettest month	mm
BIO14	Precipitation during the driest month	mm
BIO15	Precipitation seasonality	CV
BIO18	Precipitation during the warmest quarter	mm
BIO19	Precipitation during the coldest quarter	mm
ELV	Elevation	m

*Note:* Codes represent extreme or limiting environmental factors (e.g., the temperature during the coldest and warmest month and precipitation during the wet and dry quarters). A quarter is a three‐month period (1/4 of the year).

Abbreviations: °C, Centigrade degree; CV, coefficient of variation; m, meters; mm, millimeters.

Due to data points being limited and distributed unevenly across the geographical area, the MaxEnt algorithm (Phillips et al. 2006) was employed to develop ecological niche models, incorporating all available occurrences into the analysis. The models were estimated using nine principal components of the environmental variables (BIO and ELV), which accounted for 99% of the overall variance in the bioclimatic dataset. The average map model was digitized as an ASCII file (American Standard Code for Information Interchange) using ArcGIS v7.1.7.2. The MaxEnt software version 3.4.4 (Phillips et al. 2006) was used and the integrated jackknife test was employed to ascertain the effective variables for each species. The models were calibrated via 10 cross‐validation replications. In light of the inherent uncertainty of occurrence points, arising from georeferencing inaccuracies, a two‐threshold approach was employed. This approach entailed applying thresholds of 5% and 10% over the median value of the 10 replicates. This converted the model to binary predictions, where *Y* = 0 signifies a possible species presence and *Y* = 1 indicates presence points (data record). The efficacy of this technique has been demonstrated by its capacity to achieve elevated levels of predictive accuracy (Phillips and Dudík 2008). The remaining MaxEnt parameters were left at their default settings. The performance of the predictive model in each binary prediction was assessed using a jackknifing approach (Pearson et al. 2007). The model's performance was also measured in terms of area under the receiver‐operating characteristic curve (AUC, area under the curve) (Phillips et al. 2006). AUC evaluates models by assessing their capacity to differentiate between sites where a species is either ‘present’ or ‘absent’ (Phillips et al. 2006). Consequently, a model with an AUC = 0.5 exhibits a level of performance that is indistinguishable from that of a random model. Models with an AUC > 0.7 demonstrate useful performance, those with an AUC > 0.8 exhibit good performance, and those with an AUC ≥ 0.9 exhibit excellent performance (Manel et al. 2001). Additionally, MaxEnt performance was evaluated using the accuracy methods Kappa (a measure that utilizes both sensitivity and specificity) and the true skill statistic (TSS) (Allouche et al. 2006).

In instances where test data were available, the binomial probabilities were calculated exactly if the number of test samples was at most 25. In cases where this was not applicable, a normal approximation to the binomial was used. These are one‐sided *p* values for the null hypothesis that test points are predicted no better than by a random prediction with the same fractional predicted area. The “Balance” threshold is a parameter that helps to minimize the training omission rate while maintaining a certain level of accuracy. It was calculated using the formula: Balance threshold = minimizes 6 * training omission rate + 0.04 * cumulative threshold + 1.6 * fractional predicted area (Freeman and Moisen 2008; Liu et al. 2013, 2016; Merow et al. 2013).

### 2.2 Phylogenetic Reconstruction and Haplotype Analysis

The molecular dataset comprises Cytochrome *c* Oxidase subunit I (*COI*) gene sequence fragments from 221 selected *Diolcogaster* species covering a wide geographic range (Table S1). For the target *Diolcogaster* species, *D. alvearia* , *D. claritibia* , *D. mayae* , and *D. spreta* , we selected 22, 108, two, and one *COI* sequences, respectively, to determine their placement within a *COI*‐based phylogenetic tree. The published sequence data was obtained from the BOLD Systems (Barcode of Life Data System, http://www.boldsystems.org/, accessed on June 24, 2025). The sequences were registered in GenBank under accession number PX526011–PX526088. *COI* sequence alignment was performed manually. Nucleotide sequences were translated into amino acids using AliView v 1.30 (Larsson 2014) to ensure the absence of stop codons and frameshift mutations. Three microgastrine genera were used as outgroups: *Kiwigaster variabilis* Fernandez‐Triana & Ward; *Miropotes* sp.; and *Microplitis incurvatus* Xu & He, along with one Cardiochilinae species (*Cardiochiles fuscipennis* Szépligeti) and one Miracinae specimen (*Mirax* sp.). Maximum likelihood (ML) analyses were conducted using IQ‐TREE ver. 3.0.1 (Wong et al. 2025). Model selection for the IQ‐TREE analysis was performed using ModelFinder with the ModelFinder Plus (MFP) option (Kalyaanamoorthy et al. 2017), followed by an analysis with 100,000 ultrafast bootstrap (UFB) replicates (Minh et al. 2013). The best‐fitting evolutionary model was identified using ModelFinder in IQ‐TREE based on the Bayesian Information Criterion (BIC). The tree was visualized using FigTree v.1.4.4 (Rambaut 2018) and edited with Adobe Illustrator and Adobe Photoshop. *COI* sequences were analyzed to assess haplotype diversity and evolutionary distances. A TCS (Templeton, Crandall, and Sing) haplotype network (Snell et al. 2002) was constructed using the PopART program (Leigh and Bryant 2015). Prior to the analysis, the aligned sequences of the four target *Diolcogaster* species were filtered and cleaned to ensure a reliable and informative network. Several sequences were excluded due to low quality or insufficient length, as these could have introduced noise or gaps in the analysis. Only high‐quality sequences were retained to construct the TCS haplotype network and calculate genetic distances, allowing for a more accurate interpretation of haplotype structure and evolutionary relationships among the species. In the haplotype figure, single mutational changes, missing (unsampled) haplotypes, and the number of specimens sharing that specific haplotype are shown.

## 3 Results

### 3.1 Ecological Niche Modeling

**FIGURE 1:**
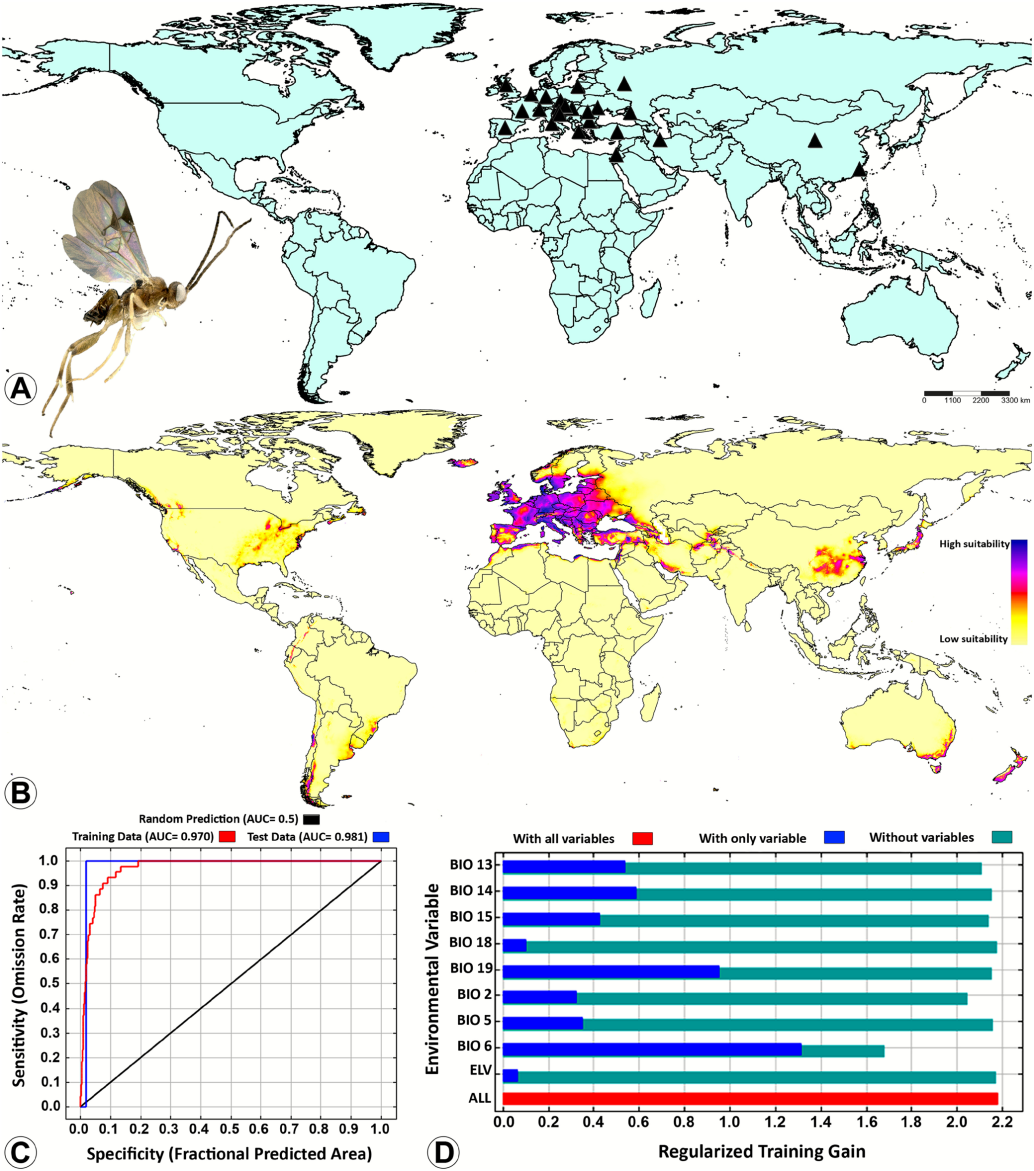
Potential suitable distributions of *Diolcogaster alvearia* (Fabricius) (Braconidae: Microgastrinae) in the current and future periods based on current climatic conditions by using the maximum entropy (MaxEnt) model. (A) Global occurrence records; (B) The predicted global suitable distribution; (C) Receiver operating characteristic (ROC) curve of potential distribution prediction; (D) Importance of the nine bioclimatic variables by Jackknife test. The habitat suitability was categorized based on the logistic output of the MaxEnt model as follows: Blue and purple = high suitability (0.75–1.0), pink and red = medium suitability (0.5–0.75), orange and yellow = low suitability (0.25–0.5), and unsuitable (0–0.25).

**FIGURE 2:**
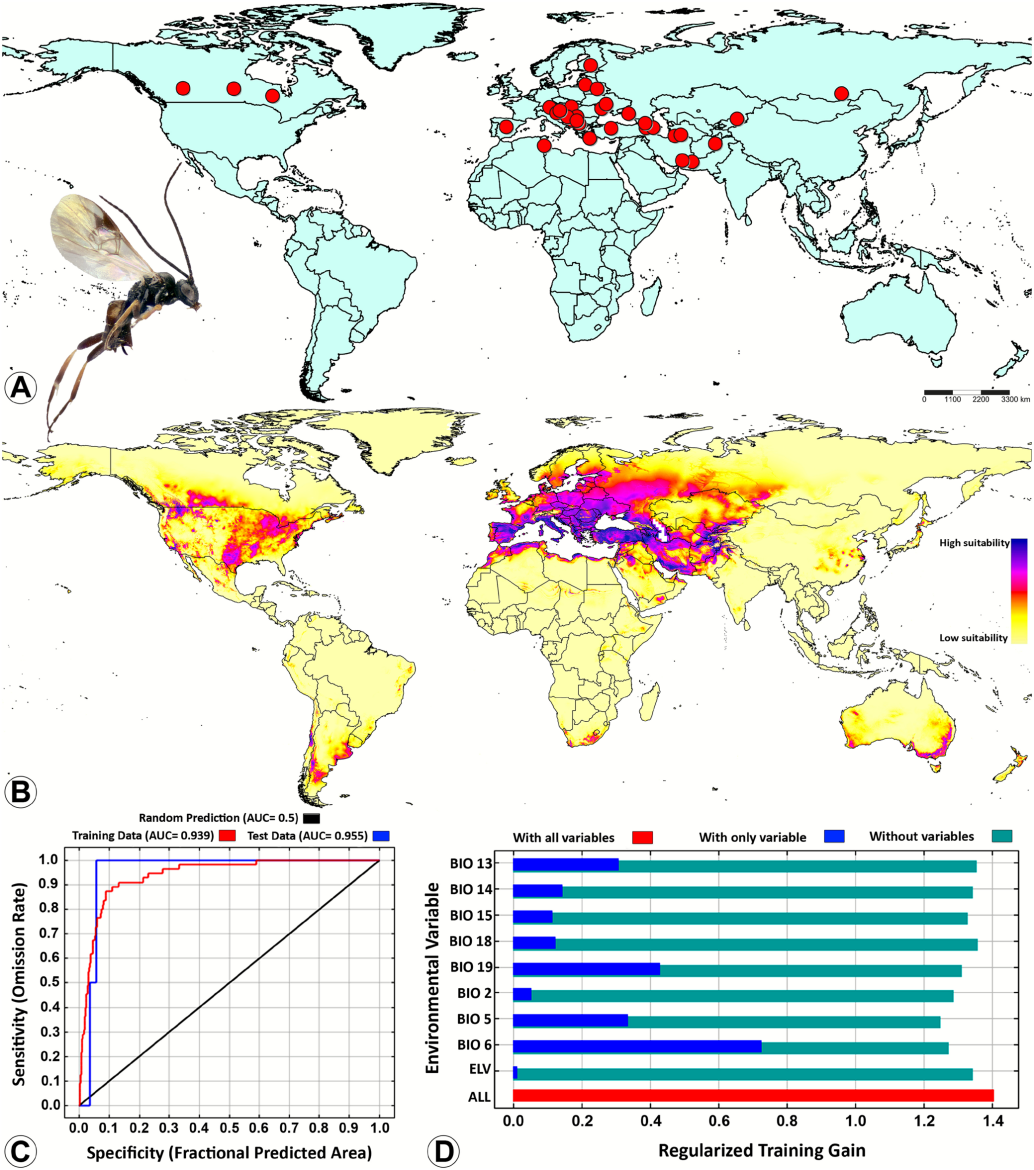
Potential suitable distributions of *Diolcogaster claritibia* (Papp) (Braconidae: Microgastrinae) in the current and future periods based on current climatic conditions by using the maximum entropy (MaxEnt) model. (A) Global occurrence records; (B) The predicted global suitable distribution; (C) Receiver operating characteristic (ROC) curve of potential distribution prediction; (D) Importance of the nine bioclimatic variables by Jackknife test. The habitat suitability was characterized based on the logistic output of the MaxEnt model as follows: Blue and purple = high suitability (0.75–1.0), pink and red = medium suitability (0.5–0.75), orange and yellow = low suitability (0.25–0.5), and unsuitable (0–0.25).

**FIGURE 3:**
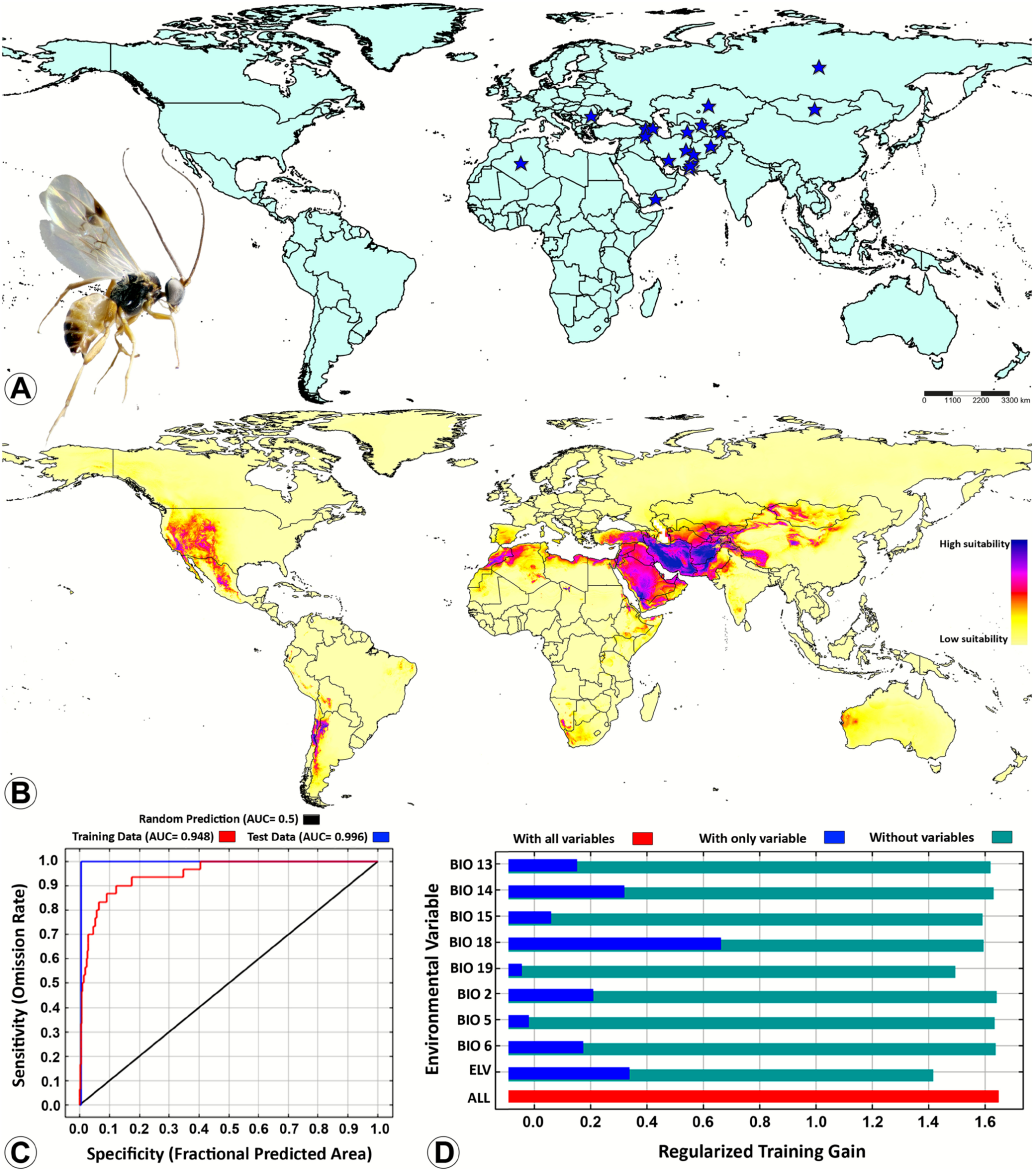
Potential suitable distributions of *Diolcogaster mayae* (Shestakov) (Braconidae: Microgastrinae) in the current and future period based on current climatic conditions by using the maximum entropy (MaxEnt) model. (A) Global occurrence records; (B) The predicted global suitable distribution; (C) Receiver operating characteristic (ROC) curve of potential distribution prediction; (D) Importance of the nine bioclimatic variables by Jackknife test. The habitat suitability was characterized based on the logistic output of the MaxEnt model as follows: Blue and purple = high suitability (0.75–1.0), pink and red = medium suitability (0.5–0.75), orange and yellow = low suitability (0.25–0.5), and unsuitable (0–0.25).

**FIGURE 4:**
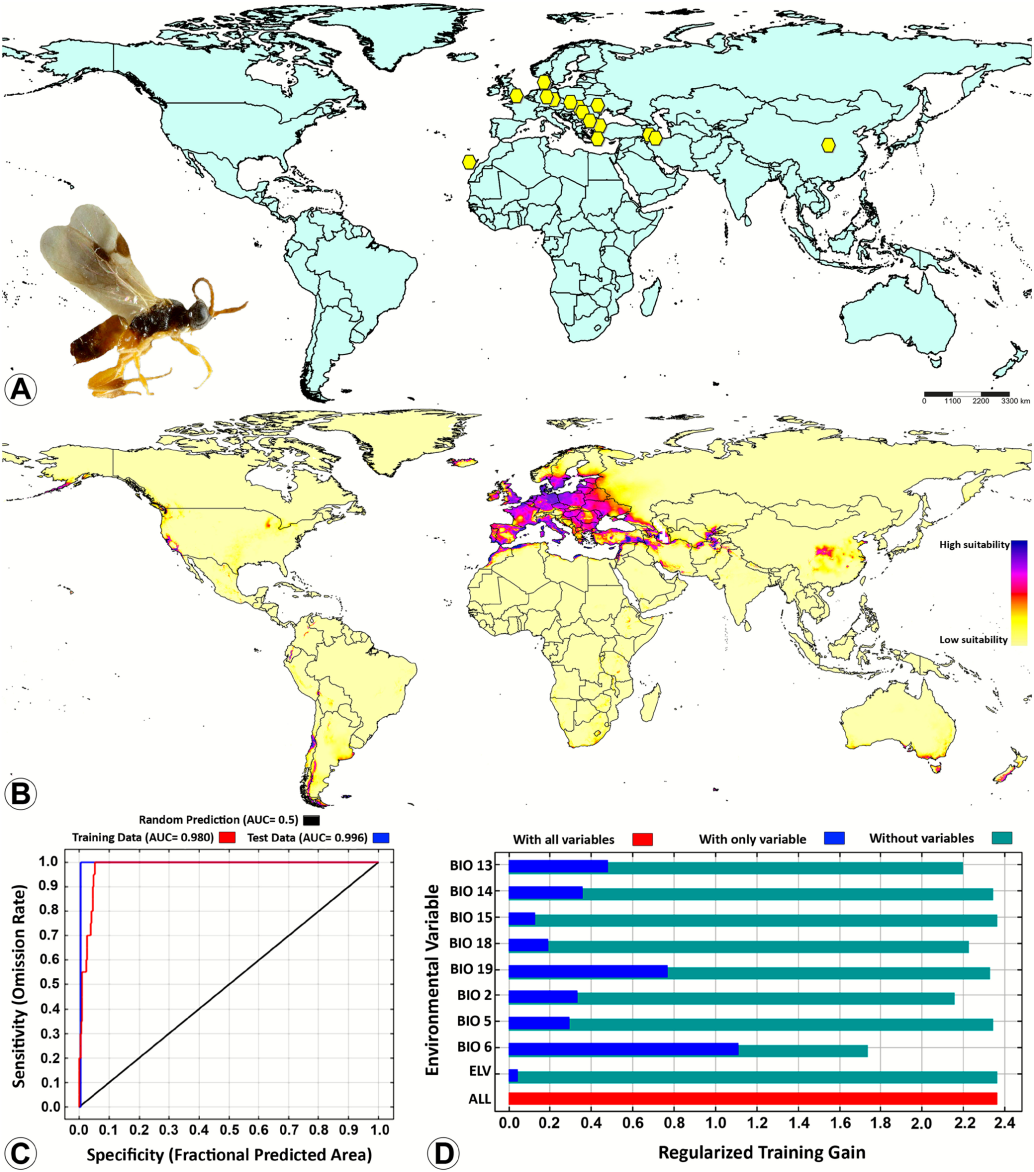
Potential suitable distributions of *Diolcogaster spreta* (Marshall) (Braconidae: Microgastrinae) in the current and future periods based on current climatic conditions by using the maximum entropy (MaxEnt) model. (A) Global occurrence records; (B) The predicted global suitable distribution; (C) Receiver operating characteristic (ROC) curve of potential distribution prediction; (D) Importance of the nine bioclimatic variables by Jackknife test. The habitat suitability was characterized based on the logistic output of the MaxEnt model as follows: Blue and purple = high suitability (0.75–1.0), pink and red = medium suitability (0.5–0.75), orange and yellow = low suitability (0.25–0.5), and unsuitable (0–0.25). The photo ‘ *D. spreta* ’ (CNCHYM 00884) has been reproduced under the terms of the Creative Commons Attribution License CC BY 4.0. The license holder is the Canadian National Collection of Insects, Arachnids, and Nematodes (CNC) in Ottawa, Canada.

The area under the curve of the training data of the MaxEnt model was 0.970 for *D. alvearia* (Figure 1C), 0.939 for *D. claritibia* (Figure 2C), 0.948 for *D. mayae* (Figure 3C), and 0.980 for *D. spreta* (Figure 4C). As these values of AUCs approach 1, the model demonstrates optimal prediction capabilities.

#### 3.1.1 Effect of the Key Environmental Variables in the Four *Diolcogaster* Species Distribution

The MaxEnt model (Table 2) indicated that the distribution of *D. alvearia* , was primarily affected by BIO6, with a percent contribution (PC) of 46.1% and having a high permutation importance (PI) of 65.7%. The other significant bioclimatic variables were BIO19 and BIO2 (Figure 1D). In the case of *D. claritibia* , BIO6 was also identified as the predominant variable with a PC of 40% and a PI of 15.2%. The other two significant variables that contributed to the model were BIO19 and BIO5. The findings indicated that *D. claritibia* is sensitive to both temperature extremes and precipitation patterns. It is noteworthy that, despite being the third variable contributing to the species distribution, BIO5 exhibited the highest PI (23.3%) among the three key BIO variables (Figure 2D). With regard to *D. mayae* , the model exhibited a significant response to BIO18, with a PC of 45.7% and a PI of 25.8%. The ELV was identified as the second most significant variable with a PC of 29% and a PI of 13.8%, suggesting that the species displays specific altitudinal preferences. BIO19 was ranked third, with a PC of 11.1% and PI of 25.8% (Figure 3D). Finally, the distribution of *D. spreta* was predominantly influenced by BIO6 with a PC of 39.1% and a remarkably elevated PI of 67.4%, which was the highest PI observed among all the *Diolcogaster* species. This finding suggests that minimum temperatures play a crucial role in determining the *D. spreta* distribution. Furthermore, BIO19 and BIO13 significantly influenced the distribution (Figure 4D).

**TABLE 2:** Percent contribution (PC) and permutation importance (PI) of the nine bioclimatic variables affecting the distribution of four species of *Diolcogaster* Ashmead (Braconidae: Microgastrinae) in the best‐fitting distribution MaxEnt model (those greater than 6% are bolded).

Code	PC/%	PI/%	Code	PC/%	PI/%
*Diolcogaster alvearia*			*Diolcogaster claritibia*		
**BIO6**	**46.1**	**65.7**	**BIO6**	**40**	**15.2**
**BIO19**	**32.4**	**3.6**	**BIO19**	**22.9**	**13.3**
**BIO2**	**11.7**	**12.2**	**BIO5**	**12**	**23.3**
**BIO13**	**6**	**10.7**	**BIO18**	**7.3**	**5**
BIO5	1.7	2.6	**BIO13**	**6.3**	**9.5**
BIO14	1.1	1.6	BIO2	5.4	9.3
BIO15	0.5	2.6	ELV	3.2	3.8
ELV	0.3	0.6	BIO15	1.8	11.6
BIO18	0.2	0.3	BIO14	1.2	9
*Diolcogaster mayae*			*Diolcogaster spreta*		
**BIO18**	**45.7**	**25.8**	**BIO6**	**39.1**	**67.4**
**ELV**	**29**	**13.8**	**BIO19**	**28.7**	**3**
**BIO19**	**11.1**	**25.8**	**BIO18**	**13.6**	**5.4**
**BIO2**	**6.3**	**1.1**	**BIO2**	**11.2**	**9**
BIO13	2.4	23.5	BIO13	5.2	12.6
BIO14	2.2	0	BIO5	1.5	1.5
BIO15	1.8	4	BIO14	0.5	0.9
BIO5	0.9	3.7	ELV	0.3	0.1
BIO6	0.7	2.3	BIO15	0	0

In summary, the BIO6 was the bioclimatic variable that most consistently influenced species distribution, especially for *D. alvearia* , *D. claritibia* , and *D. spreta* . Precipitation‐related variables, including the BIO19 and the BIO18, along with the environmental variable elevation, exert a diverse influence on distribution patterns, thereby reflecting species‐specific ecological adaptations.

The jackknife test revealed that the bioclimatic variable BIO6 had the most significant influence on the distributions of *D. alvearia* (Figure 1D), *D. claritibia* (Figure 2D), and *D. spreta* (Figure 4D). In contrast, the distribution of *D. mayae* exhibited the highest sensitivity to the bioclimatic variable BIO18 (Figure 3D). The findings indicate that BIO6 and BIO18 contribute most significantly to the observed distribution patterns. *Diolcogaster claritibia* manifests a notable degree of flexibility, demonstrating its capacity to adapt to diverse habitats, altitudes, and environmental conditions. Consequently, the distribution range of this species is likely to exceed the extant reports. This information indicates an acceptable degree of validity for the exported maps.

#### 3.1.2 Predicted Global Distribution of Suitable Habitat for Each of the Four *Diolcogaster* Species

*Diolcogaster alvearia* has been reported exclusively within the Palearctic region (Figure 1A; Fernandez‐Triana et al. 2020). However, the findings of this study, suggest that the distribution of this species in the future may extend beyond the reported region, encompassing the Australasian region (southeastern and southwestern parts of Australia and New Zealand), the Nearctic region (primarily the eastern United States, small parts of the western United States, and western Canada), and the Neotropical region (southern Brazil, Uruguay, southern and western Argentina, central Chile, and small areas of Colombia, Ecuador, and Peru) (Figure 1B). Additionally, the model predicted an extremely limited distribution of the species in the Afrotropical (South Africa) and the Oriental regions (northern India and western Nepal) (Figure 1B). Available records of herbivore hosts of *D. alvearia* are exclusively from the family Geometridae (Table 3), which is one of the most diverse lineages of Lepidoptera. The MaxEnt model predicts a wider distribution of *D. alvearia* , including the Neotropical region. This region has been observed to harbor a higher species richness of Geometridae compared to other biogeographic regions. The Neotropical region exhibits a considerably high diversity of ecosystems, including dry and humid tropical forests, cloud forests, and high‐elevation paramo (Murillo‐Ramos et al. 2021). In summary, the projected distribution of *D. alvearia* corresponds to regions characterized by a higher abundance and diversity of suitable hosts.

**TABLE 3:** List of all recorded lepidopteran hosts for the four species of *Diolcogaster* Ashmead (Braconidae: Microgastrinae) in the current study.

Species	Author	Host	Host plant	Cocoon	Larvae	MOR	MOL	BIO
*D. alvearia*	(Fabricius 1798)	Geometridae *Alcis repandata* (Linnaeus)	?	?	G	+	+	+
		*Hypomecis* sp.	*Clematis* sp. (Ranunculaceae)					
		*Menophra abruptaria* (Thunberg)	?					
		*Opisthograptis luteolata* (Linnaeus)	?					
		*Ourapteryx sambucaria* (Linnaeus)	?					
		*Peribatodes rhomboidaria* (Denis & Schiffermüller)	?					
		Undetermined Geometridae	*Laurus nobilis* L. (Lauraceae) *Fraxinus angustifolia syriaca* (Oleaceae)					
*D. claritibia*	(Papp 1959)	Plutellidae *Plutella xylostella* (Linnaeus)	*Sinapis alba* L. (Brassicaceae)	?	S	+	+	+
*D. mayae*	(Shestakov 1932)	Unknown?	?	?	?	+	+	−
*D. spreta*	(Marshall 1885)	Pyralidae *Acrobasis advenella* Zincken	?	?	S	+	+	+
		*Acrobasis consociella* (Hübner)	?					
		*Acrobasis repandana* (Fabricius)	?					
		*Pempelia palumbella* (Denis & Schiffermüller)	?					

*Note:* For references on host and host plant records, refer to Taxapad (Yu et al. 2016).

Abbreviations: ?, unknown; +, strong support; −, no support; BIO, biological data; MOL, molecular data; MOR, morphological data; G, wasp larvae gregarious; S, wasp larvae solitary.

*Diolcogaster claritibia* has only been recorded in the Holarctic region (Palearctic and Nearctic) (Figure 2A; Fernandez‐Triana et al. 2020). However, the results of this study indicated that this species may be capable of being distributed across several additional regions, encompassing the Neotropical region (Argentina, small parts of Brazil, Uruguay, and Chile), the Afrotropical region (South Africa), and the Australasian region (southeastern and southwestern parts of Australia, and New Zealand) (Figure 2B). Notably, other potential distributions were identified in certain parts of the Oriental region, specifically in Pakistan and southern India, but not in the areas of Southeast Asia (Figure 2B). The wide distribution of *D. claritibia* can be attributed to its host, *Plutella xylostella* (L.) (Lepidoptera: Plutellidae, the diamondback moth), a well‐known cosmopolitan pest (Table 3). Due to the extensive geographical distribution of this host species across multiple countries and habitats (Furlong et al. 2013; Fernandez‐Triana, Shaw, et al. 2014; Ghafouri Moghaddam et al. 2019), *D. claritibia* has adapted to diverse environmental conditions. Consequently, the MaxEnt model predicts a broad potential distribution for *D. claritibia* , consistent with its association with its globally widespread host.

*Diolcogaster mayae* has only been recorded in the Afrotropical and Palaearctic regions, with no prior reports from other biogeographical regions (Figure 3A; Fernandez‐Triana et al. 2020). However, the MaxEnt model indicates the potential for the species' presence in additional regions in the future, including the Nearctic region (eastern United States), the Neotropical region (Mexico, central Chile, eastern Argentina, and small parts of eastern Brazil, Peru, and Ecuador), the Oriental region (Pakistan, and southern and northwestern India), and the Australasian region (western Australia) (Figure 3B). Consequently, the potential for the discovery of this species in other biogeographic regions beyond its current known range is a plausible prospect. At present, no datasets are available regarding the hosts utilized by *D. mayae* (Table 3).

*Diolcogaster spreta* has been recorded exclusively in the Palaearctic region (Figure 4A; Fernandez‐Triana et al. 2020). Nonetheless, the predictive MaxEnt model suggests that this species could expand its distribution to other regions in the future under climate change and global warming. Although such potential distributions are conceivable, they appear to be feeble and constrained, yielding low suitability values, as illustrated in the map (Figure 4B). The predicted regions include the Nearctic region (the eastern and western United States, and western Canada), the Neotropical region (eastern Mexico; minor portions of Venezuela, Colombia, Ecuador, eastern Peru, and Bolivia; as well as Chile, and northern and southern Argentina), the Afrotropical region (localized areas in southern and eastern Africa), the Oriental region (northern Pakistan and India, and western Nepal), and the Australasian region (southern, southeastern, and southwestern Australia, and New Zealand) (Figure 4B). The extant lepidopteran host records are predominantly from the Pyralidae, a large and ubiquitous lepidopteran family. The documented host species have been classified into two genera from the same tribe, Phycitini (*Acrobasis* Zeller and *Pempelia* Hübner), (Table 3). These records may be indicative of a specialized tendency to attack a more limited group within the Pyralidae. The representation of the family across all geographic regions clearly underscores their adaptability and ecological importance.

In the context of current climatic conditions, *D. claritibia* (Figure 2B) is predicted to occupy the widest and the most suitable niches on a global scale, followed by *D. mayae* (Figure 3B), *D. alvearia* (Figure 1B), and finally *D. spreta* (Figure 4B). It is important to note that the optimal niches for *D. claritibia* are restricted to the western Palaearctic subregion (with a high suitability value) and the Nearctic region (with a medium suitability value) (Figure 2B).

#### 3.1.3 Response Curves of Each Individual Bioclimatic Variable and Their Predicted Suitability Habitat for the *Diolcogaster* Species

In the species *D. alvearia* (Figure S1), the BIO2 curve showed a precipitous decline in the probability of species presence as the diurnal temperature range increases. This finding suggests that the species exhibits a preference for stable daily temperatures, with its highest presence occurring at low diurnal temperatures ranging from ~2°C to 4°C. The BIO5 curve demonstrated a temperature increase, followed by a plateau, and subsequently a decline. Thus, the optimal presence of *D. alvearia* occurs roughly at moderate maximum temperatures (~20°–30°C). However, it should be noted that the actual scale may be subject to bias. The BIO6 curve was bell‐shaped, peaking at approximately –10°C–+5°C. This data suggests that *D. alvearia* is most likely present in regions that do not experience extremely cold winters. The BIO13 curve demonstrated a pronounced increase up to ~150 mm, followed by a precipitous decline. This finding suggests that the species displays a marked preference for months with moderate precipitation levels and a propensity to avoid areas with very high rainfall. The BIO14 curve showed minimal presence of the species below 50 mm, subsequently followed by an increase, and ultimately plateauing above 150 mm, with a high probability of ~0.98. The data suggest a clear preference of the species for regions that experience prolonged periods of complete dryness, with no months of precipitation. The BIO15 curve exhibited a gradual increase in species presence with increasing seasonality, peaking around 200 mm. This data suggest a propensity on the part of the species to inhabit regions characterized by significant seasonal fluctuations in rainfall. The BIO18 curve showed a precipitous decline of the species after ~100 mm, with a high probability of this decline occurring below this threshold. This finding indicates a predilection for relatively arid, temperate climates. The BIO19 curve exhibited a maximum at ~300–400 mm, followed by a decline. This data suggests that moderate precipitation during the cold season is optimal for the species. Finally, the ELV curve demonstrated a maximum probability at ~1000–1500 m, followed by a gradual decline, indicating that *D. alvearia* exhibits a preference for mid‐elevation habitats.

To summarize, the ecological preferences inferred for the MaxEnt model suggest that *D. alvearia* exhibits a preference for low daily temperature fluctuations (moderate maximum and minimum temperatures), and avoidance of extreme cold or very wet environments. The species proliferates in moderately seasonal climates characterized by mid‐level precipitation. It is likely to be found in mid‐elevation areas, such as montane forests or highland agroecosystems.

With regard to *D. claritibia* (Figure S2), the BIO2 curve demonstrated a precipitous decline in the species' presence with increasing diurnal range. The data indicate that the species is most likely to occur in regions characterized by minimal daily temperature variation (~2°C–6°C) and it has a clear preference for thermally stable microclimates. The BIO5 curve showed a direct correlation between the presence of the species and the temperature, reaching a plateau at high temperatures. This finding indicates a preference of the species for warm regions, up to a certain thermal threshold. The BIO6 curve exhibited a bell‐shaped pattern, with a maximum at approximately −10°C and +5°C, followed by a decline. The findings indicate that the species exhibits a preference for mild winters, avoiding both extreme cold and unseasonal warmth. The BIO13 curve demonstrated that the species has a high presence probability up to ~100 mm, after which there is a dramatic decline in its presence. This pattern of the curve is indicative of a predilection for regions characterized by moderately wet months with minimal rainfall. The BIO14 curve showed a marked increase up to ~50 mm, followed by a plateau with a high probability of ~0.98. This pattern suggests that the species requires consistent moisture throughout the year, exhibiting a preference for months with minimal precipitation and disfavoring extremely dry months. The BIO15 curve exhibited a pronounced increase in probability up to ~100–150 mm, followed by a slight upward plateau. This finding suggests that the species displays a preference for environments characterized by moderate to high precipitation variability throughout the year. The BIO18 curve displayed a notable presence of the species below ~100–200 mm, followed by a precipitous decline. This pattern indicates that the species has a predilection for arid, temperate climates. This preference may be associated with host activity or the synchronization of their life cycles. The BIO19 curve reached its peak at ~200–400 mm, followed by a gradual decline. The pattern suggests that moderate rainfall during the cold season is optimal for the species. Conversely, excessive rain during the cold season can lead to a decline in habitat suitability. The ELV curve demonstrates a marked increase with increasing elevation, particularly after ~1000 m, reaching a maximum at elevations greater than 5000 m. This finding suggests a strong preference for high‐elevation habitats, possibly attributable to factors such as host distribution, vegetation, or microclimate.

In summary, the ecological preferences of *D. claritibia* are areas with low diurnal variation, moderate cold winters, and high warm‐month temperatures. The species tolerates moderate rainfall during the driest and wettest months, but avoids extreme wet conditions. Furthermore, it has a notable affinity for seasonal precipitation patterns. Its association with high‐elevation environments also suggests the potential for adaptation to montane ecosystems.

In the case of *D. mayae* (Figure S3), the BIO2 curve indicated a high probability (0.77) of the species' presence at low diurnal temperatures (2°C–10°C). However, its presence exhibits a precipitous decline after 12°C. This pattern suggests a preference for environments with stable daily temperature ranges, a phenomenon that is likely driven by an aversion to significant diurnal thermal fluctuations. The BIO5 curve showed a gradual increase in probability up to very high temperatures, reaching a maximum value of 0.9. This pattern suggested tolerance and preference for elevated summer temperatures, suggesting adaptation to warm climates. The BIO6 curve exhibited a bell‐shaped pattern, peaking near −10°C to 0°C, and a concomitant decline in the presence of extreme cold or mild winters. This suggests that the species prefers mildly cold winters, avoiding both extreme frost and warmer winters. The BIO13 curve displayed a significant decrease after ~100 mm. This indicates a clear preference for regions with moderate precipitation during the wettest month and avoidance of areas with exceedingly high precipitation. The BIO14 curve showed a slight increase near 0 mm, followed by an extended period of stability (flat). The species exhibits a certain degree of tolerance for arid conditions during the dry months. However, it does not show a significant positive response to elevated precipitation during the dry season. The BIO15 curve exhibited an S‐shaped pattern which is indicative of a rapid increase up to ~150 mm, followed by a period of stability (plateau) near the maximum value of 1. This pattern suggests a strong preference for regions with distinct wet and dry seasons or high seasonality. The BIO18 curve showed an elevated presence of the species when precipitation values were near 0 mm, with a rapid, steep decline above ~200 mm. The pattern suggests a preference for dry warm seasons, possibly due to host activity or to synchronization with the host's lifecycle. The BIO19 curve exhibited a right‐skewed distribution, with a maximum probability at roughly ~150–300 mm, followed by a steady decline in probability with increasing rainfall levels. The data suggest that areas with moderately moist conditions during the cold seasons are optimal for the species' distribution, indicating a preference for conditions devoid of excessive precipitation during this period. Finally, the ELV curve showed that the species' presence diminishes below 0 m near sea level. Thereafter, the species exhibits a sharp increase and persists at a high elevation between ~1000–4000 m, with a slight decrease at higher altitudes. This indicates that the species exhibits a strong preference for environments at intermediate to high elevation, which may include mountainous regions.

In summary, the ecological profile of *D. mayae* is characterized by its affinity for low daily temperature variation, its ability to tolerate very warm summers, and its predilection for mild cold seasons. The species has a clear aversion to excessive rainfall. This species thrives under conditions of moderate wet‐season rains, very dry warm quarters, and moderate rain in the cold quarter. It also has a marked preference for seasonal climates with dry and wet periods. The species is most likely to be found in environments at mid‐ to high altitudes.

With regard to *D. spreta* (Figure S4), the BIO2 curve exhibited a half‐normal distribution. The species was present in low diurnal temperature ranges (2°C–8°C), with a high probability of ~0.8, followed by a sharp decline beyond 10°C, and the probability approached 0.0 after 19°C. The data suggest that the species prefers habitat with minimal daily temperature variability, indicating stable habitats. However, it evades regions with pronounced diurnal temperature variations. The BIO5 curve persisted with a high probability (~0.75) up to elevated temperatures. However, beyond the upper thermal range the curve precipitously declined. The species exhibits a certain degree of tolerance for moderately to very warm summer temperatures. However, it does not appear to thrive in extreme heat conditions. The BIO6 curve was bell‐shaped, peaking around −10°C–0°C, with a decline observed on both sides. The species exhibited a clear preference for moderately cold winters, and a marked aversion to both extremely cold and mild winters. This adaptation is indicative of an evolutionary response to temperate cold‐season climates. The BIO13 curve demonstrated a right‐skewed distribution, marked by a sharp increase up to ~200 mm, followed by an abrupt decline. Beyond 1000 mm of precipitation, the probability approaches zero. The species exhibited a marked preference for moderate rainfall during the wettest month of the year, and it avoids environments that are characterized by very high levels of precipitation (wets). The BIO14 curve showed a high probability of species presence at 0–50 mm of precipitation, followed by a pronounced decline beyond ~100 mm. Finally, the curve flattens above 200 mm with a minimum probability. This species is associated with dry seasons and shows a notable degree of tolerance or preference for environments with arid periods. The BIO15 variable is represented graphically as a flat line at ~0.75 probability, which persists across the entire range of values. This neutral response to precipitation seasonality, suggests that its presence is not significantly influenced by annual rainfall variability. The BIO18 curve showed its maximum probability (~1.0) at 0 mm of rain, followed by a steep decline past 300 mm; the probability of presence fell below 0.5. Finally, after 700 mm of precipitation, the curve flattened at a probability of 0. This species appears to demonstrate a clear preference for arid, temperate climates, and a marked aversion to wet warm periods. This preference could be likely associated with its lifecycle or the dynamics of its host. The BIO19 curve exhibited a high probability of species presence at near‐zero mm of precipitation, at 0.85, followed by a rapid decline until 400 mm, at ~0.45. After 400 mm, the curve flattened. The data suggest that the species prefers dry to moderately moist cold seasons, and avoids periods of heavy rain in the winter. The curve of ELV demonstrated a modest decline at sea level, followed by a gradual ascent, reaching a peak at ~6000 m. The probability exhibited a consistent rise from ~1000 m onwards. The species demonstrates a marked preference for environments situated at intermediate to elevated altitudes, with a propensity for montane or highland ecosystems. The curve showed a positive correlation, indicating that as the elevation increased, so did the probability of presence.

In summary, the ecological profile of *D. spreta* was characterized by stable daily temperatures (low diurnal range), and tolerance of high temperatures during the warm months. The species demonstrated a propensity for mildly cold winters and an aversion to both extreme frost and mild winters. The optimal precipitation level is characterized by moderate rainfall in the wettest month. The species demonstrated a high level of tolerance for extreme aridity during the driest month and warm season, indicating a preference for conditions that are less conducive to wet, warm, and cold seasons. Its response to seasonality was neutral, contrasting with the strong seasonality preference exhibited by *D. mayae* . Thus, *D. spreta* is predicted to be found in elevated regions above 1000 m, with an increasing presence toward 6000 m, suggesting an adaptation to highland or mountainous habitats.

#### 3.1.4 Predictive Accuracy of the Model Performance

A list of common thresholds and their corresponding omission rates can be found in Table S2. A detailed evaluation of the performance of the ecological niche model for *D. alvearia* , indicated that several thresholds yielded significant results (*p* < 0.05). These values indicate that the model can predict presence points with significantly greater accuracy than random selection. Among these, the “Balance” and “Equal training sensitivity and specificity” thresholds offer optimal trade‐offs between omission error and the area predicted as suitable habitat. In the case of *D. claritibia* , multiple thresholds yielded statistically significant results (*p* < 0.05), thereby indicating the model's reliable performance. Among these, the “Balance” threshold offers a practical compromise between predicted area and omission error. More conservative estimates are provided by thresholds such as “Maximum training sensitivity plus specificity” and “Equal training/test sensitivity and specificity,” which are important for minimizing false positives. Regarding *D. mayae* , none of the thresholds produced a statistically significant model (all *p* > 0.05), indicating that the predictions are no better than random. However, certain practical thresholds, such as “Fixed cumulative value 5” and “Balance training omission, predicted area, and threshold value,” still offer reasonable ecological interpretations by combining low omission rates with moderate predicted area coverage. In contrast, highly conservative thresholds (e.g., logistic threshold 0.828) are too strict, resulting in poor predictive performance. The ecological niche model for *D. spreta* demonstrates strong predictive performance. All thresholds yield statistically significant results (*p* < 0.05), confirming that the model outperforms random selection. The “Fixed cumulative value 5” (logistic threshold = 0.062, fractional predicted area = 0.140) and the “Balance training omission, predicted area and threshold value” (logistic threshold = 0.036, fractional predicted area = 0.174) are particularly useful among the tested thresholds. These thresholds maintain zero omission rates while covering a reasonable portion of the landscape, making them ecologically informative. In contrast, more conservative thresholds with high logistic values (e.g., 0.710) are overly restrictive. This results in very limited predicted areas, which could cause suitable habitats to be overlooked. Overall, the model reliably predicts the potential distribution of *D. spreta* . These thresholds are appropriate for generating binary prediction maps and supporting biogeographic interpretations of this species' potential distribution under current or future climate scenarios.

In the context of increasingly severe climate change, it has become increasingly probable that species will inhabit regions that were previously considered climatically unsuitable. Additionally, identifying new hosts for these species in these geographical regions is a plausible outcome. While such reports may initially seem unacceptable, unbelievable, or unanticipated, they are not unexpected. The utilization of ecological models in conjunction with the available information is instrumental in confirming the presence of the species and their new hosts.

### 3.2 Phylogenetic Reconstruction and Haplotype Analysis

**FIGURE 5:**
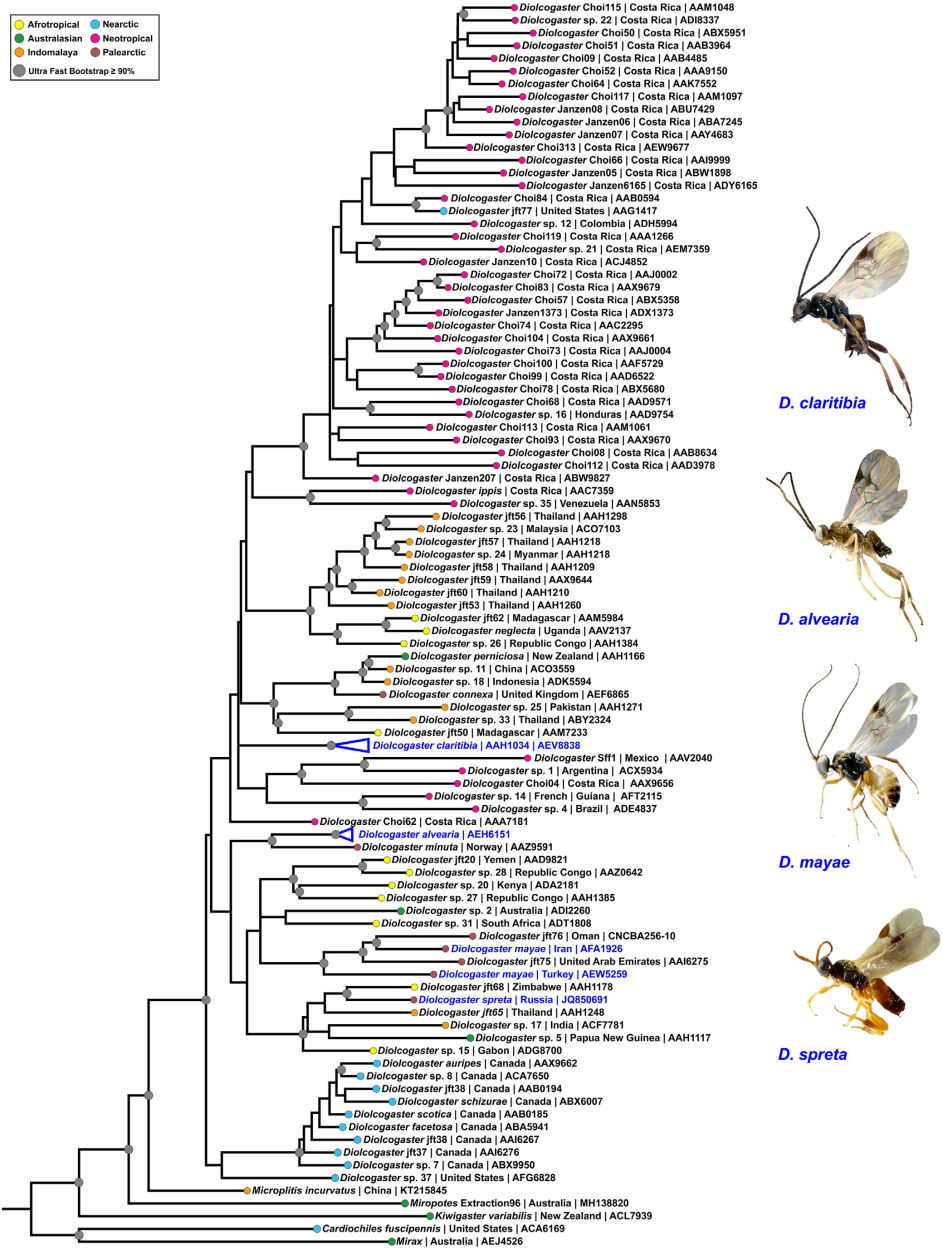
Maximum Likelihood (ML) tree to reconstruct the phylogenetic relationships of *Diolcogaster* Ashmead (Hymenoptera: Braconidae) based on *COI* data. The tree incorporates 221 *Diolcogaster* sequences from various biogeographical regions, along with *D. alvearia* (Fabricius), *D. claritibia* (Papp), *D. mayae* (Shestakov), and *D. spreta* (Marshall). These target species are indicated by a blue hue. Color codes represent different biogeographical regions: Yellow = Afrotropical; Green = Australasian; Orange = Indomalaya; Turquoise = Nearctic; Pink = Neotropical; Brown = Palaearctic. Gray nodes indicate ultrafast bootstrap support values ≥ 90%.

The *COI* phylogeny recovered well‐supported clades corresponding to the four target *Diolcogaster* species (Figure 5). Notably, none of these species constituted sister groups in a strict phylogenetic sense. Of the four species, *D. claritibia* was the most distant species and was located in a different clade than the other three species. *Diolcogaster spreta* , *D. mayae,* and *D. alvearia* were part of a different clade together, with *D. mayae* , and *D. spreta* being more closely related. *Diolcogaster claritibia* and *D. alvearia* were more genetically diverse and were phylogenetically distant from each other. All 22 *COI* sequences of *D. alvearia* clustered together. The broader clade that included *D. mayae* , *D. spreta* , and *D. alvearia* also included multiple *Diolcogaster* species from diverse regions including the Afrotropical, Australasian, Indomalayan, and Palearctic zones. Despite the fact that all 108 *COI* sequences of *D. claritibia* cluster together and form a single, well‐supported clade, the phylogenetic relationships with the other lineages within the clade remain to be determined.

**FIGURE 6:**
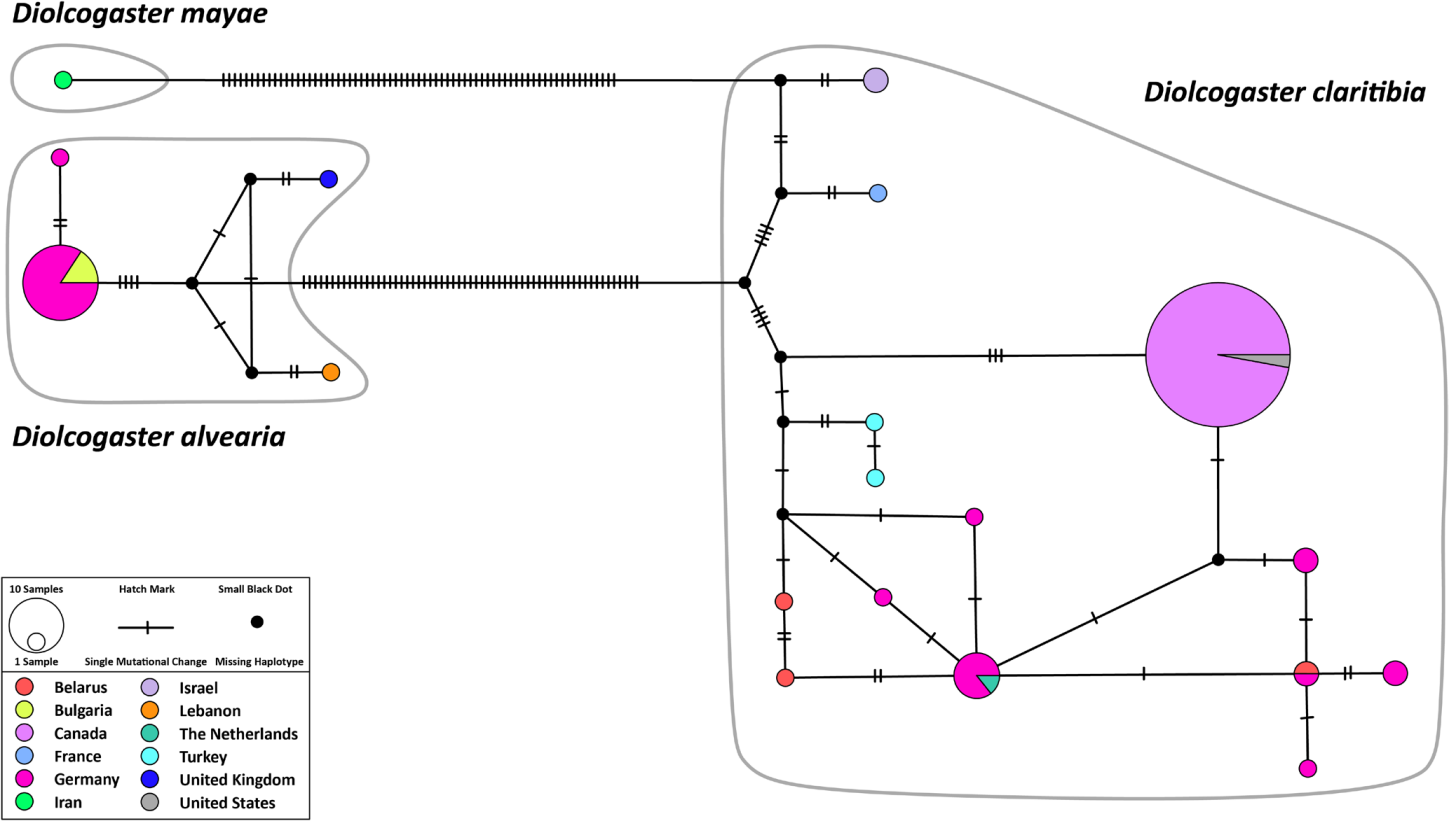
TCS haplotype network illustrating the relationships among *Diolcogaster alvearia* (Fabricius), *D. claritibia* (Papp), and *D. mayae* (Shestakov). Each circle represents a unique haplotype; the size of each circle is proportional to the number of individuals sharing that specific haplotype. Hatch marks between circles indicate the number of mutational steps separating haplotypes. Small black dots represent inferred, but unsampled (missing) haplotypes.

The TCS haplotype network (Figure 6) displayed different levels of intraspecific variation. *Diolcogaster claritibia* exhibited the highest haplotype diversity, with the presence of shared haplotypes across Europe, the Middle East, and North America. A country‐level analysis of *D. claritibia* showed a combination of both unique and shared haplotypes across distant regions. The presence of shared haplotypes in Germany, Belarus, the Netherlands, and the United Kingdom further supports the hypothesis of a stable and well‐connected population network within the Palearctic zone. Conversely, *D. alvearia* showed a reduced number of haplotypes, exhibiting a more modest geographic structure, primarily confined to Europe. Unique haplotypes were identified from Lebanon and the United Kingdom, while Germany exhibited relatively high diversity and shared haplotypes with Belarus. *Diolcogaster mayae* exhibited a single dominant haplotype, suggesting low genetic variation. The genetic diversity of *D. spreta* was found to be analogous to that observed in *D. mayae* , although this species was not included in the haplotype analysis due to poor‐quality sequences. This low genetic variation is consistent with its restricted distribution and narrow ecological niche, as predicted by ecological modeling.

## 4 Discussion

*Diolcogaster* species are well‐known parasitoid wasps that have gained widespread recognition as biological control agents against caterpillar pests (Ghafouri Moghaddam et al. 2019; Fernandez‐Triana et al. 2020). A broad‐scale understanding of their potential distribution is required for their effective implementation and utilization in agroecosystems and integrated pest management programs.

Temperature and precipitation have been identified as pivotal bioclimatic variables in shaping species distributions (Phillips et al. 2006; Hance et al. 2007; Merow et al. 2013; Furlong and Zalucki 2017; Ghafouri Moghaddam and Butcher 2023). However, the impact of specific bioclimatic variables exhibits variation among species, attributable to disparities in their developmental and ecological traits (Norberg et al. 2019; Li et al. 2022; Ghafouri Moghaddam and Butcher 2023; Drake et al. 2025). In this study, certain variables such as BIO14 and BIO15 exhibited a negligible impact on the distribution of the species *D. alvearia* , *D. claritibia* , *D. mayae* , and *D. spreta* (Table 2). While these variables may contribute to species adaptability, they appeared to have a limited impact in the examined MaxEnt model. The results of the study identified BIO6, BIO18, BIO19, and BIO2 as the key bioclimatic factors influencing the distribution of the four *Diolcogaster* species.

The response of organisms to bioclimatic variables is species‐specific. For instance, a MaxEnt‐based prediction of suitable habitats for the microgastrine wasp, *Microplitis manilae* Ashmead, identified precipitation variables (BIO13 and BIO12 = annual precipitation), and temperature variables (BIO1 = annual mean temperature, BIO4 = temperature seasonality, and BIO10 = mean temperature during the warmest quarter), as the most influential factors (e.g., Ghafouri Moghaddam and Butcher 2023). This underscores the necessity of conducting species‐specific analyses when assessing the role of bioclimatic factors. The approach and findings presented in this study can serve as a valuable reference and comparative framework for modeling the distribution of other parasitoid wasp species.

The predictive MaxEnt model revealed that none of the four *Diolcogaster* species examined in this study demonstrated potential distribution in Southeast Asia or Madagascar (Figures 1B, 2B, 3B, 4B). The phenomenon can be explained by several tentative reasons. Firstly, the unique climatic and ecological conditions in these regions such as consistently high temperatures, extreme humidity, and dense tropical vegetation (Hending et al. 2022; Ma et al. 2025) may not align with the environmental preferences of these four species. Secondly, the high degree of specialization and diversity in the wasp faunas of Southeast Asia and Madagascar suggests intense interspecific competition (Songvorawit et al. 2021; Butcher and Quicke 2023; Goodman 2023; Ghafouri Moghaddam, Quicke, et al. 2025; Lu et al. 2025). This competition may hinder the successful establishment of species introduced from other locations or those with distinct ecological characteristics. Thirdly, host availability and habitat specificity are important factors to consider (Novotny and Basset 2005; Koch 2021; Braga and Janz 2021), as the host species utilized by *Diolcogaster* species in other regions may be rare, absent, or replaced by unrelated taxa in these areas. Finally, historical biogeographic isolation and limited natural dispersal pathways (Wiens and Donoghue 2004) may have impeded the colonization of Southeast Asia and Madagascar by these *Diolcogaster* species.

The prediction of the global suitable distribution results of this study serves as a useful reference for assessing the potential distribution of the four *Diolcogaster* species in various regions. Presently, documentation of these species is predominantly concentrated within the Palearctic region (Figures 1A, 2A, 3A, 4A). However, MaxEnt model predictions (Figures 1B, 2B, 3B, 4B) indicate that suitable habitats under present climate conditions extend across multiple countries in different biogeographical regions, suggesting a broader potential distribution. A multitude of critical factors must be taken into consideration when introducing these parasitoids into new areas. These factors encompass, but are not limited to, the host specificity, the identity of the herbivore host, the species origin (native or introduced), and the genetic variability (e.g., Ghafouri Moghaddam, Tomlinson, et al. 2022; Ghafouri Moghaddam, Arias‐Penna, et al. 2022; Ghafouri Moghaddam and Butcher 2023). The present study is exclusively concerned with the influence of climatic variables on species distribution. An in‐depth evaluation of all pertinent ecological and biological factors is critical to ensure the effective, safe, and sustainable management of caterpillar pests through biological control.

Due to the dearth of host species data for specific *Diolcogaster* species under scrutiny, such as *D. mayae* (Table 3; Yu et al. 2016; Ghafouri Moghaddam et al. 2019), ascertaining its precise distributions remains impracticable. Nonetheless, the MaxEnt ecological model simulations predict that the distribution of this species is projected to extend to specific temperate, subtropical, and tropical regions. *Diolcogaster mayae* was characterized by the model as a highly adaptable species with the ability to thrive in diverse environmental conditions (Figure 3B). This finding suggests the potential presence of suitable herbivore host species in these regions. Consequently, further information, including host species and biological data, is necessary to validate these predictions (De la Pérez et al. 2020; Ghafouri Moghaddam and Butcher 2023). Notwithstanding, these models will furnish researchers with significant preliminary insights to guide their search for hosts of this species. The identification of these potential regions is particularly significant, as it enables researchers to focus their field research efforts and develop a more efficient strategy for discovering previously unknown or overlooked host species.

The *COI*‐based phylogenetic analysis provides unequivocal genetic delimitation among the four *Diolcogaster* species (Figure 5). The absence of immediate sister‐group relationships among the species indicates significant evolutionary divergence. The deeper genetic splits and higher haplotype diversity observed in *D. claritibia* (Figure 6) are consistent with its broad ecological tolerance and extensive geographic distribution across both the Palearctic and Nearctic regions (Figure 2B). These findings align with the hypothesis that more widespread taxa tend to accumulate greater genetic variation over time. The phylogenetic relationships of *D. claritibia* with other lineages within the clade remain unresolved (Figure 5). The observed polytomy may be attributable to several factors, including limited phylogenetic signal of the *COI* marker, incomplete lineage sorting, or insufficient taxon sampling (Mardulyn and Whitfield 1999; Whitfield et al. 2002; Banks and Whitfield 2006; Ghafouri Moghaddam, Quicke, et al. 2025). Such unresolved nodes are common in mitochondrial‐based phylogenies of microgastrine parasitoid wasps, where rapid radiations and conservative mitochondrial evolution can obscure deeper relationships (Banks and Whitfield 2006; Ghafouri Moghaddam, Quicke, et al. 2025). Nevertheless, *D. claritibia* , a Holarctic species with a broad distribution, may represent an older or more stable lineage within the genus. *Diolcogaster spreta* and *D. mayae* exhibited low intraspecific genetic variation across sampled populations (Figure 6) and showed limited range expansions (Figures 3B, 4B), suggesting limited historical dispersal, recent range expansion, or potential demographic bottlenecks. However, based on the predicted MaxEnt models, *D. mayae* appears to be ecologically versatile, with the capacity to adapt to diverse habitats, elevations, and environmental conditions (Figure 3B,D). Given the paucity of molecular data for this species, further sampling and genetic analysis will most likely reveal patterns consistent with the ecological predictions of the MaxEnt models. *Diolcogaster alvearia* , a species that is geographically restricted to the western Palearctic region (Figure 1A), serves as an example of a potential pattern of regional lineage diversification. *Diolcogaster alvearia* formed a well‐supported clade with *D. minuta* , another western Palearctic species (Figure 5), supporting a biogeographic clustering pattern. The distribution of *D. alvearia* may extend beyond the currently reported region in the future, as indicated by both the MaxEnt models and molecular analyses (Figures 1B, 5, 6). The inclusion of *D. mayae* , *D. spreta* , and *D. alvearia* in a clade with species from multiple biogeographic regions highlights the possibility that *Diolcogaster* harbors substantial cryptic or undiscovered diversity worldwide. This broader pattern suggests that the genus encompasses considerable unrecognized diversity and complex biogeographic histories.

The haplotype results for *D. claritibia* suggest two potential evolutionary scenarios: either high gene flow across continents or a recent range expansion. The presence of unique haplotypes in countries such as Israel, France, Turkey, Canada, and the United States (Figure 6) supports the hypothesis that *D. claritibia* is a genetically versatile species with a high capacity for ecological adaptation. The elevated level of haplotype diversity observed in *D. claritibia* (Figure 6), together with the presence of both unique and shared haplotypes across distant regions, corresponds with the broader ecological niches predicted by the ENMs (Figure 2B), indicating high dispersal ability and potential for local adaptation. In contrast, the restricted range and minimal genetic diversity in *D. spreta* and *D. mayae* (Figure 6) imply a more limited ecological amplitude or a recent colonization of the sampled areas. These patterns support the hypothesis that haplotype richness may serve as a proxy for adaptive potential under shifting climatic conditions (Saccheri and Hanski 2006; Lanfear et al. 2014). Given the potential for range expansion, *D. claritibia* and *D. mayae* may be superior candidates for biocontrol programs in new or changing agroecosystems. The congruence between ecological models and molecular data reinforces their resilience to environmental change. The monitoring of haplotype dynamics over time can facilitate the tracking of expansion fronts and the detection of potential local adaptation (Sherpa and Després 2021; Bernatchez et al. 2024).

A substantial body of research has demonstrated that global climate change exerts a considerable influence on the distribution patterns of numerous species (Heidari Latibari, Moravvej, Arias‐Penna, et al. 2022; Rubenstein et al. 2023; Drake et al. 2025). The findings of this study indicate that climate change may result in regional or local declines, and potentially even extinction, of certain *Diolcogaster* species in specific areas. In order to provide support for the conservation and effective use of these parasitoid wasps, three key recommendations are proposed herewith:

**Protected species and introduced species are the target.** The protection of existing species or the introduction of foreign species as biological control agents must be based on the predicted suitability under varying climate conditions. Furthermore, the environmental and ecological conditions of each region must be taken into consideration.

**Monitoring and adaptive rearing.** It is crucial to establish continuous monitoring and tracking mechanisms to assess the establishment of these species in the field. Furthermore, the development of adaptive rearing strategies that align with both model predictions and real‐world observations is essential, particularly in areas where suitable habitats are anticipated to undergo changes.

**Strengthen systematic research.** In order to enhance the accuracy and success of rearing and establishment programs, it is essential to enhance both morphological and molecular studies of *Diolcogaster* species. Current research on these species remains limited; therefore, advancing taxonomic and genetic knowledge is critical for effective management and conservation.

## Author Contributions

**Asma Moeinadini:** conceptualization (equal), formal analysis (equal), data curation (equal), investigation (equal), methodology (equal), validation (equal), visualization (equal), writing – original draft (equal). **Mostafa Ghafouri Moghaddam:** conceptualization (equal), formal analysis (equal), data curation (equal), investigation (equal), methodology (equal), validation (equal), visualization (equal), writing – original draft (equal). **Diana Carolina Arias‐Penna:** investigation (equal), writing – review and editing (equal). **Seyed Massoud Madjdzadeh:** investigation (equal), writing – review and editing (equal). **Minoo Heidari Latibari:** formal analysis (equal), data curation (equal), investigation (equal), validation (equal), visualization (equal), writing – review and editing (equal). **Buntika A. Butcher:** conceptualization (supporting), funding acquisition (equal), investigation (equal), project administration (equal), resources (equal), supervision (equal), visualization (equal), writing – review and editing (equal).

## Ethics Statement

The authors have nothing to report.

## Consent

The authors have nothing to report.

## Conflicts of Interest

The authors declare no conflicts of interest.

## Supporting information


**Figure S1:** Response curves of the nine bioclimatic variables and its predicted suitability for *Diolcogaster alvearia* (Fabricius) (Hymenoptera: Braconidae, Microgastrinae).

**Figure S2:** Response curves of the nine bioclimatic variables and its predicted suitability for *Diolcogaster claritibia* (Papp) (Hymenoptera: Braconidae, Microgastrinae).

**Figure S3:** Response curves of the nine bioclimatic variables and its predicted suitability for *Diolcogaster mayae* (Shestakov) (Hymenoptera: Braconidae, Microgastrinae).

**Figure S4:** Response curves of the nine bioclimatic variables and its predicted suitability for *Diolcogaster spreta* (Marshall) (Hymenoptera: Braconidae, Microgastrinae).

**Table S1:** List of the 221 *Diolcogaster* (Hymenoptera: Braconidae, Microgastrinae) specimens included in the analysis of mitochondrial cytochrome *c* oxidase subunit I (*COI*), along with five outgroups. For each specimen, codes, depositories, and places of origin are provided.
**Table S2:** List of common thresholds and their corresponding omission rates for the model's predictive accuracy. A total of four species of *Diolcogaster* Ashmead (Hymenoptera: Braconidae, Microgastrinae) were used in the study.

## Data Availability

The data that supports the findings of this study are available in the Supporting Information of this article.

## References

[ece372547-bib-0001] Allouche, O. , A. Tsoar , and R. Kadmon . 2006. “Assessing Species Distribution Models' Accuracy: Prevalence, Kappa, and the True Skill Statistic (TSS).” Journal of Applied Ecology 43, no. 6: 1223–1232. 10.1111/j.1365-2664.2006.01214.x.

[ece372547-bib-0002] Arias‐Penna, D. C. , J. B. Whitfield , D. H. Janzen , et al. 2019. “A Species‐Level Taxonomic Review and Host Associations of *Glyptapanteles* (Hymenoptera, Braconidae, Microgastrinae) With an Emphasis on 136 New Reared Species From Costa Rica and Ecuador.” ZooKeys 890: 1–685. 10.3897/zookeys.890.35786. 31798309 PMC6881475

[ece372547-bib-0003] Arias‐Penna, D. C. , J. B. Whitfield , D. H. Janzen , and W. Hallwachs . 2013. “Three New Species in the Genus *Wilkinsonellus* (Braconidae, Microgastrinae) From the Neotropics, and the First Host Record for the Genus.” ZooKeys 302: 79–95. 10.3897/zookeys.302.4962. PMC3689143 23794899

[ece372547-bib-0004] Banks, J. C. , and J. B. Whitfield . 2006. “Dissecting the Ancient Rapid Radiation of Microgastrine Wasp Genera Using Additional Nuclear Genes.” Molecular Phylogenetics and Evolution 41, no. 3: 690–703. 10.1016/j.ympev.2006.06.001. 16854601 PMC7129091

[ece372547-bib-0005] Bernatchez, L. , A. L. Ferchaud , C. S. Berger , C. J. Venney , and A. Xuereb . 2024. “Genomics for Monitoring and Understanding Species Responses to Global Climate Change.” Nature Reviews Genetics 25, no. 3: 165–183. 10.1038/s41576-023-00657-y. 37863940

[ece372547-bib-0006] Braga, M. P. , and N. Janz . 2021. “Host Repertoires and Changing Insect–Plant Interactions.” Ecological Entomology 46, no. 6: 1241–1253. 10.1111/een.13073.

[ece372547-bib-0007] Branca, A. , B. Le Ru , F. Vavre , J.‐F. Silvain , and S. Dupas . 2011. “Intraspecific Specialization of the Generalist Parasitoid *Cotesia sesamiae* Revealed by polyDNAvirus Polymorphism and Associated With Different *Wolbachia* Infections.” Molecular Ecology 20: 959–971. 10.1111/j.1365-294X.2010.04977.x. 21255170

[ece372547-bib-0008] Butcher, B. A. , and D. L. J. Quicke . 2023. The Parasitoid Wasps of South East Asia, 967. CAB International.

[ece372547-bib-0009] Castex, V. , M. Beniston , P. Calanca , D. Fleury , and J. Moreau . 2018. “Pest Management Under Climate Change: The Importance of Understanding Tritrophic Relations.” Science of the Total Environment 616: 397–407. 10.1016/j.scitotenv.2017.11.027. 29127793

[ece372547-bib-0010] Dalton, R. M. , N. C. Underwood , D. W. Inouye , M. E. Soulé , and B. D. Inouye . 2023. “Long‐Term Declines in Insect Abundance and Biomass in a Subalpine Habitat.” Ecosphere 14, no. 8: e4620. 10.1002/ecs2.4620.

[ece372547-bib-0011] De la Pérez, O. N. B. , S. Espinosa‐Zaragoza , V. López‐Martínez , D. S. Hight , and L. Varone . 2020. “Ecological Niche Modeling to Calculate Ideal Sites to Introduce a Natural Enemy: The Case of *Apanteles opuntiarum* (Hymenoptera: Braconidae) to Control *Cactoblastis cactorum* (Lepidoptera: Pyralidae) in North America.” Insects 11, no. 7: 454. 10.3390/insects11070454. 32707668 PMC7411794

[ece372547-bib-0012] Drake, J. M. , J. P. Wares , J. E. Byers , and J. T. Anderson . 2025. “Two Hypotheses About Climate Change and Species Distributions.” Ecology Letters 28, no. 5: e70134. 10.1111/ele.70134. 40344332 PMC12061546

[ece372547-bib-0013] Dupas, S. , C. Gitau W. , A. Branca , B. Le Ru , and J. F. Silvain . 2008. “Evolution of a Polydnavirus Gene in Relation to Parasitoid‐Host Species Immune Resistance.” Journal of Heredity 99: 491–499. 10.1093/jhered/esn047. 18552349

[ece372547-bib-0014] Fernandez‐Triana, J. , M. R. Shaw , C. Boudreault , M. Beaudin , and G. R. Broad . 2020. “Annotated and Illustrated World Checklist of Microgastrinae Parasitoid Wasps (Hymenoptera, Braconidae).” ZooKeys 920: 1–1089. 10.3897/zookeys.920.39128. 32390740 PMC7197271

[ece372547-bib-0015] Fernandez‐Triana, J. , M. R. Shaw , S. Cardinal , L. Dosdall , and P. Mason . 2014. “First Nearctic Record of *Diolcogaster claritibia* (Hymenoptera: Braconidae: Microgastrinae), With Notes on Taxonomic Status and Natural History.” Canadian Entomologist 146: 609–620. 10.4039/tce.2014.16.

[ece372547-bib-0016] Fernández‐Triana, J. , J. B. Whitfield , J. J. Rodriguez , et al. 2014. “Review of *Apanteles* Sensu Stricto (Hymenoptera: Braconidae, Microgastrinae) From Area de Conservación Guanacaste, Northwestern Costa Rica, With Keys to All Described Species From Mesoamerica.” ZooKeys 383: 1–565. 10.3897/zookeys.383.6418. PMC3950464 24624021

[ece372547-bib-0017] Freeman, E. A. , and G. G. Moisen . 2008. “A Comparison of the Performance of Threshold Criteria for Binary Classification in Terms of Predicted Prevalence and Kappa.” Ecological Modelling 217, no. 1–2: 48–58. 10.1016/j.ecolmodel.2008.05.015.

[ece372547-bib-0018] Furlong, M. J. , D. J. Wright , and L. M. Dosdall . 2013. “Diamondback Moth Ecology and Management: Problems, Progress, and Prospects.” Annual Review of Entomology 58: 517–541. 10.1146/annurev-ento-120811-153605. 23020617

[ece372547-bib-0019] Furlong, M. J. , and M. P. Zalucki . 2017. “Climate Change and Biological Control: The Consequences of Increasing Temperatures on Host–Parasitoid Interactions.” Current Opinion in Insect Science 20: 39–44. 10.1016/j.cois.2017.03.006. 28602234

[ece372547-bib-0020] Galli, M. , F. Feldmann , U. K. Vogler , and K.‐H. Kogel . 2024. “Can Biocontrol Be the Game‐Changer in Integrated Pest Management? A Review of Definitions, Methods and Strategies.” Journal of Plant Diseases and Protection 131: 265–291. 10.1007/s41348-024-00878-1.

[ece372547-bib-0021] Ghafouri Moghaddam, M. , D. C. Arias‐Penna , and M. Heidari Latibari . 2022. “Notes on the Genus *Choeras* Mason, 1981 (Hymenoptera: Ichneumonoidea, Braconidae, Microgastrinae) From Iran.” Tijdschrift Voor Entomologie 165: 37–48. 10.1163/22119434-bja10021.

[ece372547-bib-0022] Ghafouri Moghaddam, M. , and B. A. Butcher . 2023. “ *Microplitis manilae* Ashmead (Hymenoptera: Braconidae): Biology, Systematics, and Response to Climate Change Through Ecological Niche Modelling.” Insects 14, no. 4: 338. 10.3390/insects14040338. 37103153 PMC10143999

[ece372547-bib-0023] Ghafouri Moghaddam, M. , J. L. Fernandez‐Triana , and D. Ward . 2025. “Microgastrinae Wasps of the World.” http://microgastrinae.myspecies.info/.

[ece372547-bib-0024] Ghafouri Moghaddam, M. , D. L. Quicke , D. C. Arias‐Penna , et al. 2025. “Expansion of the Distributional Range of *Qrocodiledundee* Fernández‐Triana (Hymenoptera: Braconidae: Microgastrinae), With the Description of a New Species in the Oriental Region Using an Integrative Taxonomy Approach.” Integrative Systematics: Stuttgart Contributions to Natural History 8, no. 1: 11–34. 10.18476/2025.978354.

[ece372547-bib-0026] Ghafouri Moghaddam, M. , E. Rakhshani , C. van Achterberg , and A. Mokhtari . 2018. “A Study of the Iranian Species of *Choeras* Mason (Hymenoptera: Braconidae: Microgastrinae), With the Description of a New Species.” Zootaxa 4446, no. 4: 455–476. 10.11646/zootaxa.4446.4.3. 30313870

[ece372547-bib-0027] Ghafouri Moghaddam, M. , E. Rakhshani , C. van Achterberg , and A. Mokhtari . 2019. “A Taxonomic Review of the Genus *Diolcogaster* Ashmead (Hymenoptera, Braconidae, Microgastrinae) in Iran, Distribution and Morphological Variability.” Zootaxa 4590, no. 1: 95–124. 10.11646/zootaxa.4590.1.4 31716102

[ece372547-bib-0025] Ghafouri Moghaddam, M. , E. Rakhshani , C. van Achterberg , and A. Mokhtari . 2021. “Revision of the Genus *Napamus* Papp (Hymenoptera, Braconidae, Microgastrinae).” International Journal of Tropical Insect Science 41: 2529–2542. 10.1007/s42690-021-00433-7.

[ece372547-bib-0028] Ghafouri Moghaddam, M. , S. Tomlinson , S. Jaffe , et al. 2022. “Review of the New World Species of *Microplitis* Foerster (Hymenoptera, Braconidae, Microgastrinae) Attacking Sphingidae (Lepidoptera, Bombycoidea).” Insect Systematics and Evolution 53, no. 3: 221–241. 10.1163/1876312X-bja10026.

[ece372547-bib-0029] Gitau, C. W. , D. Gundersen‐Rindal , M. Pedroni , P. J. Mbugi , and S. Dupas . 2007. “Differential Expression of the CrV1 Haemocyte Inactivation‐Associated Polydnavirus Gene in the African Maize Stem Borer *Busseola fusca* (Fuller) Parasitized by Two Biotypes of the Endoparasitoid *Cotesia sesamiae* (Cameron).” Journal of Insect Physiology 53: 676–684. 10.1016/j.jinsphys.2007.04.008. 17570392

[ece372547-bib-0030] Gitau, C. W. , F. Schulthess , and S. Dupas . 2010. “An Association Between Host Acceptance and Virulence Status of Different Populations of *Cotesia sesamiae* , a Braconid Larval Parasitoid of Lepidopteran Cereal Stemborers in Kenya.” Biological Control 54: 100–106. 10.1016/j.biocontrol.2010.04.010.

[ece372547-bib-0031] Goodman, S. M. 2023. “Updated Estimates of Biotic Diversity and Endemism for Madagascar—Revisited After 20 Years.” Oryx 57: 561–565. 10.1017/S0030605322001284.

[ece372547-bib-0032] Guisan, A. , W. Thuiller , and N. E. Zimmermann . 2017. Habitat Suitability and Distribution Models: With Applications in R Ecology, Biodiversity and Conservation. Cambridge University Press.

[ece372547-bib-0033] Hance, T. , J. van Baaren , P. Vernon , and G. Boivin . 2007. “Impact of Extreme Temperatures on Parasitoids in a Climate Change Perspective.” Annual Review of Entomology 52, no. 1: 107–126. 10.1146/annurev.ento.52.110405.091333. 16846383

[ece372547-bib-0034] Harvey, J. A. , K. Tougeron , R. Gols , et al. 2023. “Scientists' Warning on Climate Change and Insects.” Ecological Monographs 93: ecm.1553. 10.1002/ecm.1553.

[ece372547-bib-0035] Hayhoe, K. E. , J. Edmonds , R. Kopp , A. LeGrande , and B. Sanderson . 2017. “Climate Models, Scenarios, and Projections.” Publications, Agencies and Staff of the U.S. Department of Commerce. 589. https://digitalcommons.unl.edu/usdeptcommercepub/58.

[ece372547-bib-0036] Heidari Latibari, M. , D. C. Arias‐Penna , M. Ghafouri Moghaddam , and B. A. Butcher . 2025. “Bacterial Symbiont as Game Changers for *Aphis craccivora* Koch's Fitness and Survival Across Distinct Climate Types.” Scientific Reports 15: 14208. 10.1038/s41598-025-98690-w. 40269010 PMC12019319

[ece372547-bib-0037] Heidari Latibari, M. , G. Moravej , M. Ghafouri Moghaddam , H. Barahoei , and G. A. Hanley . 2020. “The Novel Host Associations for the Aphid Parasitoid, *Pauesia hazratbalensis* (Hymenoptera: Braconidae: Aphidiinae).” Oriental Insects 54: 88–95. 10.1080/00305316.2019.1586780.

[ece372547-bib-0038] Heidari Latibari, M. , G. Moravvej , D. C. Arias‐Penna , and M. Ghafouri Moghaddam . 2022. “Effects of Carbon Monoxide, Nitrogen Dioxide, and Fine Particulate Matter on Insect Abundance and Diversity in Urban Green Spaces.” Scientific Reports 12: 17574. 10.1038/s41598-022-20577-x. 36284111 PMC9596448

[ece372547-bib-0040] Heidari Latibari, M. , G. Moravvej , E. Rakhshani , J. Karimi , and D. C. Arias‐Penna . 2022. “A Host Record for a Strictly Specific Aphid Parasitoid *Aphidius smithi* (Braconidae: Aphidiinae): The Food Plant‐Host Aphid‐Parasitoid Association Puzzle Acquires a New Piece.” Biocontrol Scence and Technology 32: 1389–1402. 10.1080/09583157.2022.2124234.

[ece372547-bib-0039] Heidari Latibari, M. , G. Moravvej , E. Rakhshani , J. Karimi , D. C. Arias‐Penna , and B. A. Butcher . 2023. “ *Arsenophonus*: A Double‐Edged Sword of Aphid Defense Against Parasitoids.” Insects 14, no. 9: 763. 10.3390/insects14090763. 37754731 PMC10531911

[ece372547-bib-0041] Hending, D. , M. Holderied , G. McCabe , and S. Cotton . 2022. “Effects of Future Climate Change on the Forests of Madagascar.” Ecosphere 13, no. 4: e4017. 10.1002/ecs2.4017.

[ece372547-bib-0042] Hijmans, R. J. , S. E. Cameron , J. L. Parra , P. G. Jones , and A. Jarvis . 2005. “Very High‐Resolution Interpolated Climate Surfaces for Global Land Areas.” International Journal of Climatology 25: 1965–1978. 10.1002/joc.1276.

[ece372547-bib-0043] Kaiser, L. , B. P. Le Ru , F. Kaoula , et al. 2015. “Ongoing Ecological Speciation in *Cotesia sesamiae*, a Biological Control Agent of Cereal Stem Borers.” Evolutionary Applications 8: 807–820. 10.1111/eva.12260. 26366198 PMC4561570

[ece372547-bib-0044] Kalyaanamoorthy, S. , B. Q. Minh , T. K. F. Wong , A. von Haeseler , and L. S. Jermiin . 2017. “ModelFinder: Fast Model Selection for Accurate Phylogenetic Estimates.” Nature Methods 14: 587–589. 10.1038/nmeth.4285. 28481363 PMC5453245

[ece372547-bib-0045] Koch, F. H. 2021. “Considerations Regarding Species Distribution Models for Forest Insects.” Agricultural and Forest Entomology 23, no. 4: 393–399. 10.1111/afe.12458.

[ece372547-bib-0046] Kogan, M. 1998. “Integrated Pest Management: Historical Perspectives and Contemporary Developments.” Annual Review of Entomology 43: 243–270. 10.1146/annurev.ento.43.1.243. 9444752

[ece372547-bib-0047] Lanfear, R. , H. Kokko , and A. Eyre‐Walker . 2014. “Population Size and the Rate of Evolution.” Trends in Ecology & Evolution 29, no. 1: 33–41. 10.1016/j.tree.2013.09.009. 24148292

[ece372547-bib-0048] Larsson, A. 2014. “AliView: A Fast and Lightweight Alignment Viewer and Editor for Large Data Sets.” Bioinformatics 30, no. 22: 3276–3278. 10.1093/bioinformatics/btu531. 25095880 PMC4221126

[ece372547-bib-0049] Leigh, J. W. , and D. Bryant . 2015. “PopART: Full‐Feature Software for Haplotype Network Construction.” Methods in Ecology and Evolution 6, no. 9: 1110–1116. 10.1111/2041-210X.12410.

[ece372547-bib-0050] Li, D. , Z. Li , Z. Liu , et al. 2022. “Climate Change Simulations Revealed Potentially Drastic Shifts in Insect Community Structure and Crop Yields in China's Farmland.” Journal of Pest Science 96: 55–69. 10.1007/s10340-022-01479-3.

[ece372547-bib-0051] Liu, C. , G. Newell , and M. White . 2016. “On the Selection of Thresholds for Predicting Species Occurrence With Presence‐Only Data.” Ecology and Evolution 6, no. 1: 337–348. 10.1002/ece3.1878. 26811797 PMC4716501

[ece372547-bib-0052] Liu, C. , M. White , and G. Newell . 2013. “Selecting Thresholds for the Prediction of Species Occurrence With Presence‐Only Data.” Journal of Biogeography 40, no. 4: 778–789. 10.1111/jbi.12058.

[ece372547-bib-0053] Lu, G. , S. Chen , Y. Lei , et al. 2025. “Taxonomic Study of the Genus *Diolcogaster* Ashmead (Hymenoptera, Braconidae, Microgastrinae) From Borneo With the Description of Four New Species.” PLoS One 20, no. 10: e0332071. 10.1371/journal.pone.0332071. 41091698 PMC12527212

[ece372547-bib-0054] Ma, Z. , H. Liu , and Y. Yang . 2025. “Impacts of Global Climate Change on the Spatial Range of Insects Distributed Across the Pacific Ocean.” Ecological Informatics 90: 103247. 10.1016/j.ecoinf.2025.103247.

[ece372547-bib-0055] Manel, S. , H. C. William , and S. J. Ormerod . 2001. “Evaluating Presence‐Absence Models in Ecology: The Need to Account for Prevalence.” Journal of Applied Ecology 38, no. 5: 291–931. 10.1046/j.1365-2664.2001.00647.x.

[ece372547-bib-0056] Mardulyn, P. , and J. B. Whitfield . 1999. “Phylogenetic Signal in the *COI*, *16S*, and *28S* Genes for Inferring Relationships Among Genera of Microgastrinae (Hymenoptera; Braconidae): Evidence of a High Diversification Rate in This Group of Parasitoids.” Molecular Phylogenetics and Evolution 12, no. 3: 282–294. 10.1006/mpev.1999.0618. 10413623

[ece372547-bib-0057] Menéndez, R. , A. González‐Megías , Y. Collingham , et al. 2007. “Direct and Indirect Effects of Climate and Habitat Factors on Butterfly Diversity.” Ecology 88, no. 3: 605–611. 10.1890/06-0539. 17503588

[ece372547-bib-0058] Merow, C. , M. J. Smith , and J. A. Silander Jr. 2013. “A Practical Guide to MaxEnt for Modeling Species' Distributions: What It Does, and Why Inputs and Settings Matter.” Ecography 36, no. 10: 1058–1069. 10.1111/j.1600-0587.2013.07872.x.

[ece372547-bib-0059] Minh, B. Q. , M. A. T. Nguyen , and A. von Haeseler . 2013. “Ultra‐Fast Approximation for Phylogenetic Bootstrap.” Molecular Biology and Evolution 30, no. 5: 1188–1195. 10.1093/molbev/mst024. 23418397 PMC3670741

[ece372547-bib-0061] Murillo‐Ramos, L. , P. Sihvonen , G. Brehm , I. C. Ríos‐Malaver , and N. Wahlberg . 2021. “A Database and Checklist of Geometrid Moths (Lepidoptera) From Colombia.” Biodiversity Data Journal 9: e68693. 10.3897/BDJ.9.e68693. 34566452 PMC8433126

[ece372547-bib-0062] Norberg, A. , N. Abrego , F. G. Blanchet , et al. 2019. “A Comprehensive Evaluation of Predictive Performance of 33 Species Distribution Models at Species and Community Levels.” Ecological Monographs 89: e01370.

[ece372547-bib-0063] Novotny, V. , and Y. Basset . 2005. “Host Specificity of Insect Herbivores in Tropical Forests.” Proceedings of the Royal Society B: Biological Sciences 272, no. 1568: 1083–1090. 10.1098/rspb.2004.3023. PMC1559807 16024368

[ece372547-bib-0064] Olson, D. M. , E. Dinerstein , E. D. Wikramanayake , et al. 2001. “Terrestrial Ecoregions of the World: A New Map of Life on Earth.” Bioscience 51: 933–938. 10.1641/0006-3568(2001)051[0933:TEOTWA]2.0.CO;2.

[ece372547-bib-0065] Outhwaite, C. L. , P. McCann , and T. Newbold . 2022. “Agriculture and Climate Change Are Reshaping Insect Biodiversity Worldwide.” Nature 605: 97–102. 10.1038/s41586-022-04644-x. 35444282

[ece372547-bib-0066] Parmesan, C. , and G. Yohe . 2003. “A Globally Coherent Fingerprint of Climate Change Impacts Across Natural Systems.” Nature 421, no. 6918: 37–42. 10.1038/nature01286. 12511946

[ece372547-bib-0067] Pearson, R. G. , C. J. Raxworthy , M. Nakamura , and A. T. Peterson . 2007. “Predicting Species Distributions From Small Numbers of Occurrence Records: A Test Case Using Cryptic Geckos in Madagascar.” Journal of Biogeography 34: 102–117. 10.1111/j.1365-2699.2006.01594.x.

[ece372547-bib-0068] Peshin, R. , and A. K. Dhawan . 2009. Integrated Pest Management: Innovation‐Development Process. Springer Netherlands. 10.1007/978-1-4020-8992-3.

[ece372547-bib-0069] Phillips, S. J. , R. P. Anderson , and R. E. Schapire . 2006. “Maximum Entropy Modeling of Species Geographic Distributions.” Ecological Modelling 190: 231–259. 10.1016/j.ecolmodel.2005.03.026.

[ece372547-bib-0070] Phillips, S. J. , and M. Dudík . 2008. “Modeling of Species Distributions With Maxent: New Extensions and a Comprehensive Evaluation.” Ecography 31, no. 2: 161–175. 10.1111/j.0906-7590.2008.5203.x.

[ece372547-bib-0071] Rambaut, A. 2018. “FigTree, v.1.4.4.” Computer Program Distributed by the Author. http://tree.bio.ed.ac.uk/software/figtree/.

[ece372547-bib-0072] Ramos Aguila, L. C. , X. Li , K. S. Akutse , et al. 2023. “Host–Parasitoid Phenology, Distribution, and Biological Control Under Climate Change.” Life 13: 2290. 10.3390/life13122290. 38137891 PMC10744521

[ece372547-bib-0073] Rubenstein, M. A. , S. R. Weiskopf , R. Bertrand , et al. 2023. “Climate Change and the Global Redistribution of Biodiversity: Substantial Variation in Empirical Support for Expected Range Shifts.” Environmental Evidence 12: 7. 10.1186/s13750-023-00296-0. 39294691 PMC11378804

[ece372547-bib-0074] Saccheri, I. , and I. Hanski . 2006. “Natural Selection and Population Dynamics.” Trends in Ecology & Evolution 21, no. 6: 341–347. 10.1016/j.tree.2006.03.018. 16769435

[ece372547-bib-0075] Shaw, M. R. 2012. “Notes on Some European Microgastrinae (Hymenoptera: Braconidae) in the National Museums of Scotland, With Twenty Species New to Britain, New Host Data, Taxonomic Changes and Remarks, and Descriptions of Two New Species of *Microgaster* Latreille.” Entomologist's Gazette 63, no. 3: 173–201.

[ece372547-bib-0076] Sherpa, S. , and L. Després . 2021. “The Evolutionary Dynamics of Biological Invasions: A Multi‐Approach Perspective.” Evolutionary Applications 14, no. 6: 1463–1484. 10.1111/eva.13215. 34178098 PMC8210789

[ece372547-bib-0077] Skendžić, S. , M. Zovko , I. P. Živković , V. Lešić , and D. Lemić . 2021. “The Impact of Climate Change on Agricultural Insect Pests.” Insects 12: 440. 10.3390/insects12050440. 34066138 PMC8150874

[ece372547-bib-0078] Snell, Q. , P. Walker , D. Posada , and K. Crandall . 2002. “TCS: Estimating Gene Genealogies.” International Parallel and Distributed Processing Symposium 2: 184–191.

[ece372547-bib-0079] Sofaer, H. R. , J. J. Barsugli , C. S. Jarnevich , et al. 2017. “Designing Ecological Climate Change Impact Assessments to Reflect Key Climatic Drivers.” Global Change Biology 23: 2537–2553. 28173628 10.1111/gcb.13653

[ece372547-bib-0080] Songvorawit, N. , D. Quicke , and B. A. Butcher . 2021. “Taxonomic Progress and Diversity of Ichneumonoid Wasps (Hymenoptera: Ichneumonoidea) in Southeast Asia.” Tropical Natural History 21, no. 1: 78–93. 10.58837/tnh.21.1.248068.

[ece372547-bib-0081] Spahn, R. , and J. T. Lill . 2022. “Higher Temperatures Reduce the Efficacy of a Key Biocontrol Parasitoid.” Biological Control 176: 105079. 10.1016/j.biocontrol.2022.105079.

[ece372547-bib-0082] Thomson, L. J. , S. Macfadyen , and A. A. Hoffmann . 2010. “Predicting the Effects of Climate Change on Natural Enemies of Agricultural Pests.” Biological Control 52: 296–306. 10.1016/j.biocontrol.2009.01.022.

[ece372547-bib-0083] Tokpah, D. P. , H. Li , L. Wang , X. Liu , Q. S. Mulbah , and H. Liu . 2016. “An Assessment System for Screening Effective Bacteria as Biological Control Agents Against Magnaporthe Grisea on Rice.” Biological Control 103: 21–29. 10.1016/j.biocontrol.2016.07.009.

[ece372547-bib-0084] Tougeron, K. , J. Brodeur , C. Le Lann , and J. van Baaren . 2019. “How Climate Change Affects the Seasonal Ecology of Insect Parasitoids.” Ecological Entomology 45, no. 2: 167–181. 10.1111/een.12792.

[ece372547-bib-0085] Trew, B. T. , and I. M. Maclean . 2021. “Vulnerability of Global Biodiversity Hotspots to Climate Change.” Global Ecology and Biogeography 30, no. 4: 768–783. 10.1111/geb.13272.

[ece372547-bib-0086] Urbina‐Cardona, N. , M. E. Blair , M. C. Londoño , R. Loyola , J. Velásquez‐Tibatá , and H. Morales‐Devia . 2019. “Species Distribution Modeling in Latin America: A 25‐Year Retrospective Review.” Tropical Conservation Science 12: 194008291985405.

[ece372547-bib-0087] Valerio, A. A. , and J. B. Whitfield . 2015. “Taxonomic Review of the Genus *Hypomicrogaster* Ashmead (Hymenoptera: Braconidae: Microgastrinae), With Descriptions of 40 New Species.” Zootaxa 3979, no. 1: 1–98. 10.11646/zootaxa.3979.1.1. 26249935

[ece372547-bib-0088] Whitfield, J. B. , A. Austin , and J. L. Fernandez‐Triana . 2018. “Systematics, Biology, and Evolution of Microgastrine Parasitoid Wasps.” Annual Review of Entomology 63: 389–406. 10.1146/annurev-ento-020117-043405. 29058979

[ece372547-bib-0089] Whitfield, J. B. , P. Mardulyn , A. D. Austin , and M. Dowton . 2002. “Phylogenetic Relationships Among Microgastrine Braconid Wasp Genera Based on Data From the *16S*, *COI* and *28S* Genes and Morphology.” Systematic Entomology 27, no. 3: 337–359. 10.1046/j.1365-3113.2002.00183.x.

[ece372547-bib-0090] Wiens, J. J. , and M. J. Donoghue . 2004. “Historical Biogeography, Ecology and Species Richness.” Trends in Ecology & Evolution 19, no. 12: 639–644. 10.1016/j.tree.2004.09.011. 16701326

[ece372547-bib-0091] Wong, T. K. , N. Ly‐Trong , H. Ren , et al. 2025. “IQ‐TREE 3: Phylogenomic Inference Software Using Complex Evolutionary Models.” https://ecoevorxiv.org/repository/view/8916/. 10.1093/molbev/msag117 PMC13191116 42085559

[ece372547-bib-0092] Yu, D. S. K. , C. van Achterberg , and K. Horstmann . 2016. “Taxapad 2016, Ichneumonoidea 2015.” Database on Flash‐Drive: Nepean, Ontario.

[ece372547-bib-0093] Žikić, V. , M. Mitrović , S. Stanković , et al. 2024. “An Integrative Taxonomic Study of North Temperate *Cotesia* Cameron (Hymenoptera, Braconidae, Microgastrinae) That Form Silken Cocoon Balls, With the Description of a New Species.” Journal of Hymenoptera Research 97: 255–276. 10.3897/jhr.97.116378.

