# Supplementary figures and images for: Predicting Potential Range Shifts and Molecular Approaches in Four Diolcogaster Ashmead Species (Hymenoptera: Braconidae, Microgastrinae)
Source: Ecol Evol. 2025 Nov 26;15(12):e72547. doi: 10.1002/ece3.72547 (PMC12657638; doi:10.1002/ece3.72547)

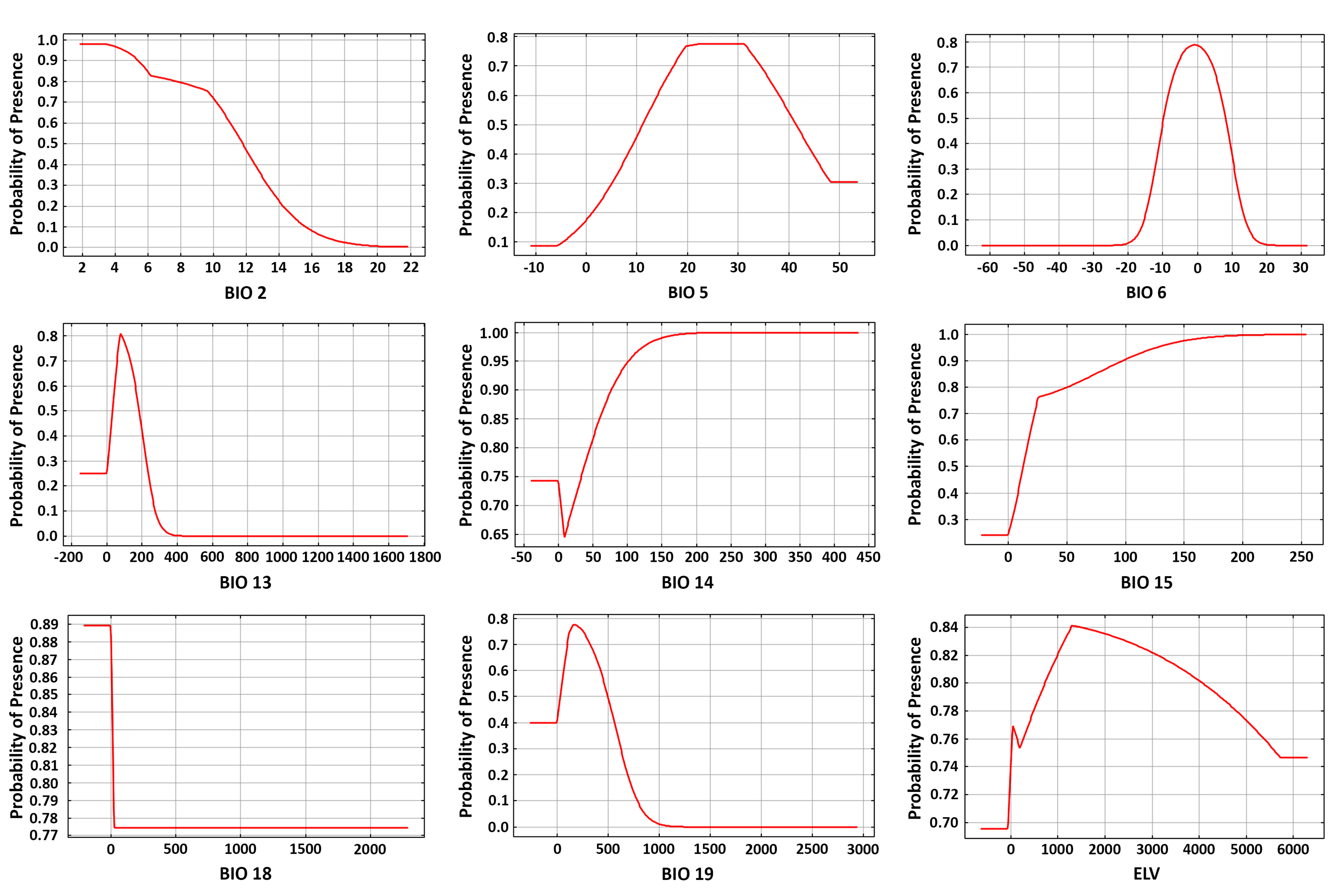

Supplement: Supplementary file 1 — Figure S1: Response curves of the nine bioclimatic variables and its predicted suitability for Diolcogaster alvearia (Fabricius) (Hymenoptera: Braconidae, Microgastrinae). [file ECE3-15-e72547-s005.png]

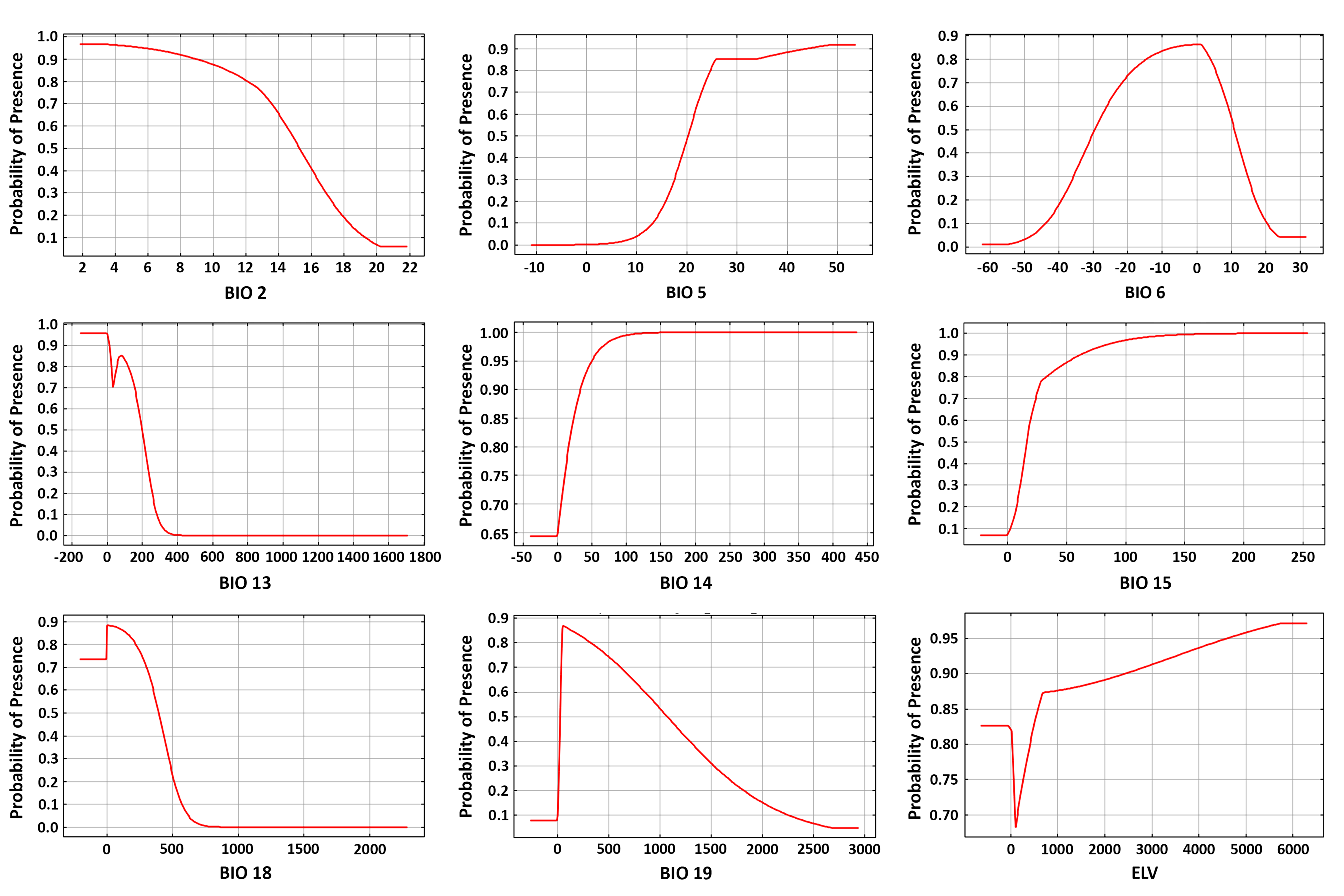

Supplement: Supplementary file 2 — Figure S2: Response curves of the nine bioclimatic variables and its predicted suitability for Diolcogaster claritibia (Papp) (Hymenoptera: Braconidae, Microgastrinae). [file ECE3-15-e72547-s001.png]

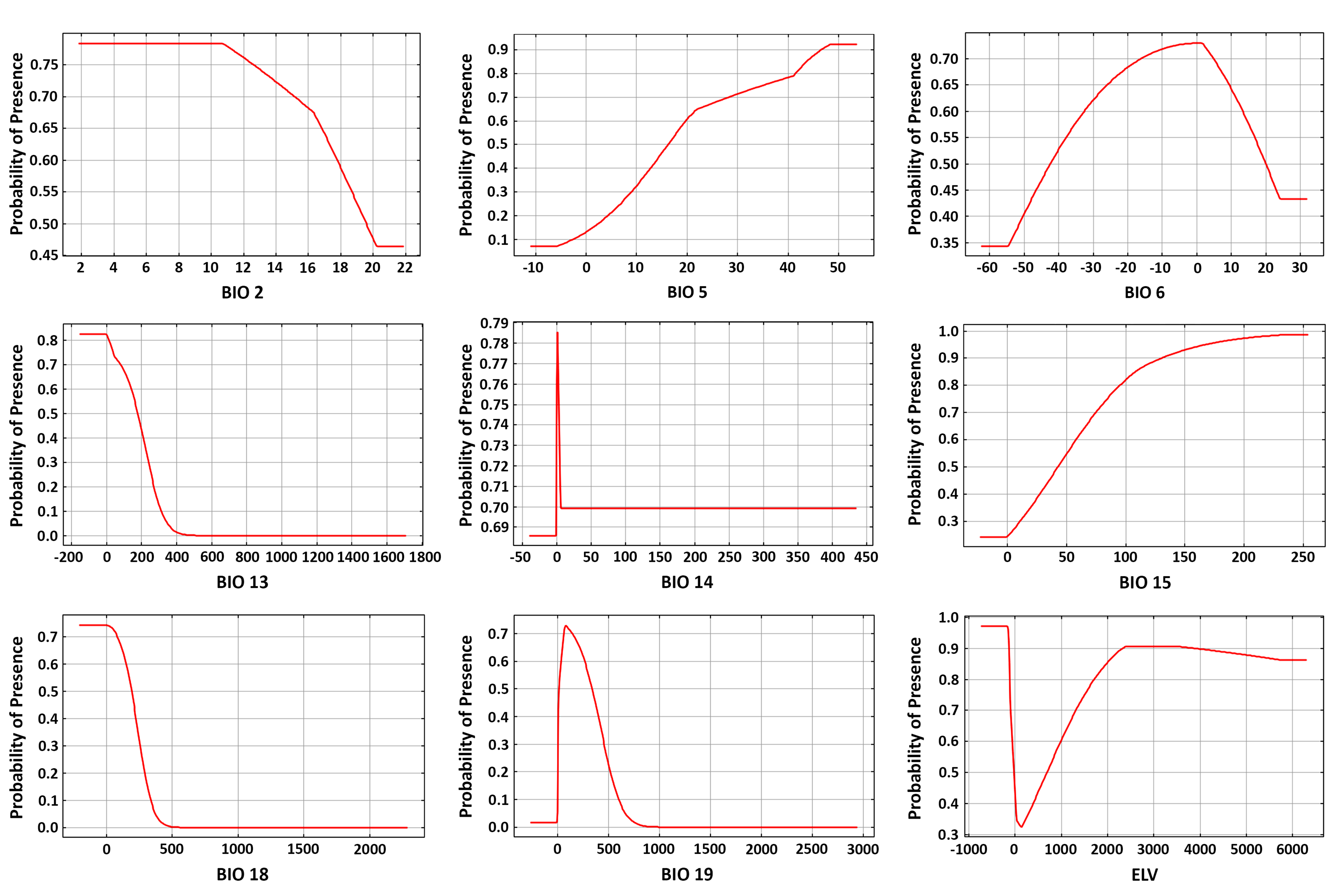

Supplement: Supplementary file 3 — Figure S3: Response curves of the nine bioclimatic variables and its predicted suitability for Diolcogaster mayae (Shestakov) (Hymenoptera: Braconidae, Microgastrinae). [file ECE3-15-e72547-s003.png]

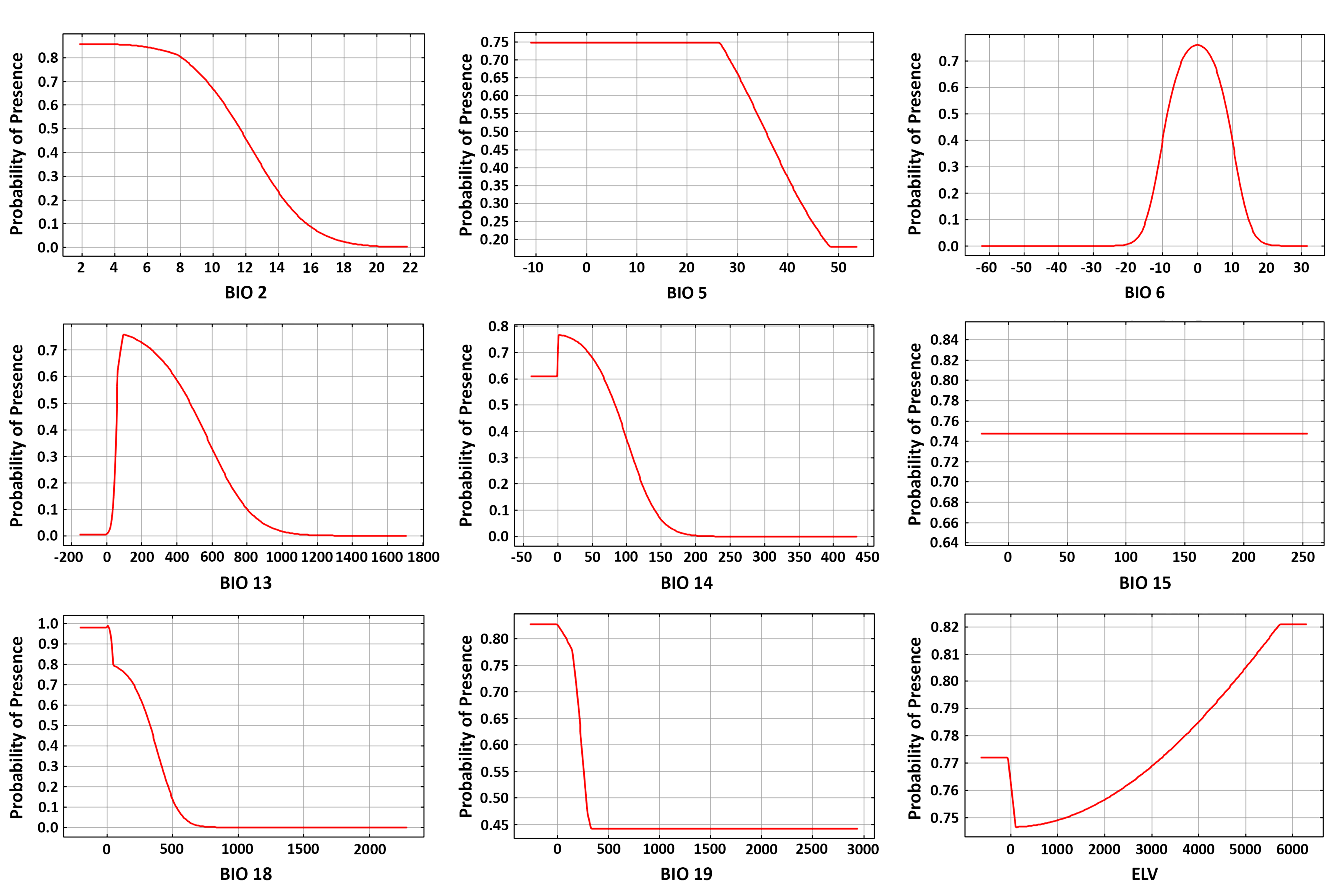

Supplement: Supplementary file 4 — Figure S4: Response curves of the nine bioclimatic variables and its predicted suitability for Diolcogaster spreta (Marshall) (Hymenoptera: Braconidae, Microgastrinae). [file ECE3-15-e72547-s002.png]
